# Trehalose: a multifunctional sugar and its metabolites are biotechnological targets for crop salinity tolerance under saline conditions

**DOI:** 10.1007/s11103-026-01705-x

**Published:** 2026-04-23

**Authors:** Mohamed Magdy F. Mansour, Fahmy A. S. Hassan

**Affiliations:** 1 Department of Botany, Faculty of Science, Ain Shams University, Cairo, 11566 Egypt; 2 Department of Horticulture, Faculty of Agriculture, Tanta University, Tanta, 31527 Egypt

**Keywords:** Biotechnological strategies, Pivotal functions, Salinity stress, Trehalose, Trehalose metabolites

## Abstract

Salinity stress is an abiotic threat that impairs crop growth, development, and productivity, placing a heavy burden on global agriculture. Understanding how crops tolerate salinity enables the development of biotechnological strategies to enhance crop resilience and yield, thereby securing sustainable food supplies for the growing global population. Trehalose, its metabolite trehalose-6-phosphate (T6P), along with key biosynthetic enzymes (trehalose-6-phosphate synthase, TPS; trehalose-6-phosphate phosphatase, TPP), have garnered increasing attention for their ability to enhance crop tolerance to salinity by modulating physiological, biochemical, and signaling processes. Therefore, strategies to increase trehalose levels or to enhance its metabolic functions are promising for researchers seeking to improve crop tolerance and yield in saline environments. This review explores the structure of trehalose, its biosynthesis, protective molecular mechanisms, and the vital functions of its metabolites in enhancing crop tolerance and productivity in saline soils. It also underscores biotechnological strategies targeting trehalose metabolism to enhance salinity resilience of crops, while critically evaluating current limitations and knowledge gaps. Further targeted manipulation of trehalose metabolism, tissue-specific regulations, and validating results in field-grown crops are also required to translate laboratory findings into agricultural applications.

## Key message

Recent advances have been presented, demonstrating that trehalose and its metabolites perform diverse roles under saline conditions, making them promising biomolecules for biotechnological approaches to crop improvement.

## Introduction

Agriculture provides the food and raw materials needed for human nutrition and many economic industries, making it a crucial part of the world economy. Unfortunately, biotic and abiotic stresses pose numerous hazards to global agriculture, which is not keeping pace with the escalating global population, placing a higher burden on food demands worldwide. Salinity stress is an abiotic constraint that restricts plant growth, development, distribution, and productivity. Approximately one billion hectares of global arable land have been severely impacted by salinity over the last decade, and it is expected to result in a 50% loss by 2050 (FAO 2009). Excess salts in the soil, due to their osmotic stress, can adversely reduce plant water uptake, cellular turgor pressure, and expansion. Long-term exposure to salinity results in the accumulation of toxic ions, leading to plant ion toxicity and nutrient imbalance, commonly referred to as ionic stress caused by salinity. Additionally, salinity stress leads to increased ROS production, which damages membrane lipids, proteins, and nucleic acids (Hasanuzzaman et al. 2021; Kesawat et al. 2023). These hazardous effects of salinity cause drastic modifications in the morphological, physiological, biochemical, and molecular functions of plants, with these effects being less severe in salinity-tolerant crop plants/genotypes. (Atta et al. 2023; Harsha et al. 2025; Shahid et al. 2020).

**Fig. 1:**
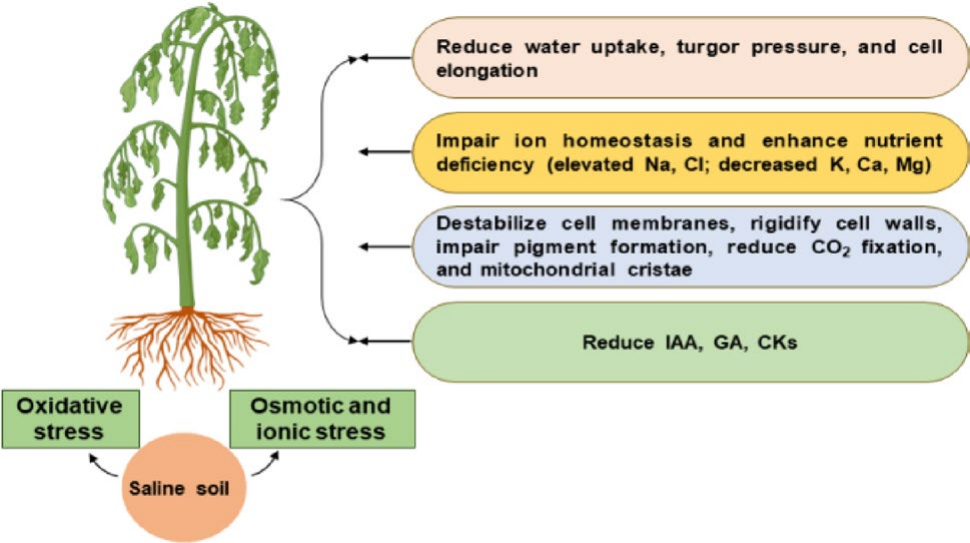
Salinity stress harmful impacts (osmotic, ionic, oxidative stresses) result in crop growth, development, and yield reduction

Salt-resistant crop plants cope with salinity stress through various mechanisms: osmotic adjustment, ion homeostasis, antioxidant defense systems, and membrane and cell wall modifications (Mansour and Salama 2019; Zhang et al. 2022b; Atta et al. 2023). Figure 1 summarizes the harmful effects of salinity stress that reduce crop yield. Mitigating the negative impacts of salinity stress and understanding how crop plants tolerate salinity are crucial for increasing agricultural productivity and food security. Tolerant crop plants exposed to salinity stress synthesize and accumulate large amounts of secondary metabolites that serve signaling functions, buffer cellular redox potential, act as chaperones, serve as a storage form of nitrogen and carbon, induce ROS detoxifying systems, and stabilize cellular macromolecules (Mansour and Salama 2019, 2020; Ghosh et al. 2021; Atta et al. 2023; Mansour 2023; Sarkar and Sadhukhan 2022; Ibrahim 2025). Notably, these metabolites mediate osmotic adjustment when their absolute concentrations are high, but at low levels, they show signaling and protective functions under stress (Eh et al. 2015; Han et al. 2024). Additionally, several methods are used to reduce the damaging effects of high salinity: biotechnological approaches using advanced genetic engineering and non-genetic strategies that involve applying natural plant metabolites externally (Mansour 2023). Recent genetic studies, along with metabolome and transcriptome analyses, have identified various candidate genes for salinity tolerance that should be used in genetic engineering efforts to improve crop resilience to saline environments (Goharrizi et al. 2022; Zhu et al. 2023; Atta et al. 2023). As climate change is expected to worsen adverse conditions, using these metabolome biomarkers in agricultural research provides a promising method to develop crops with improved salinity stress tolerance. These crops can grow efficiently and produce high yields even under challenging conditions.

Evidence suggests that crop plants can produce trehalose, one of the frequently accumulated soluble sugars under salinity stress, and it was originally believed to serve as an osmoprotectant (Grennan 2007; Iturriaga et al. 2009). However, the presence of trehalose in plants at low levels (0.1-2 nmol g^− 1^ FW) makes its role as an osmoprotectant unlikely (Eh et al. 2025). A growing body of evidence indicates its involvement in plant growth and development, numerous signaling cascades and metabolic pathways, ion homeostasis, and its vital role in stimulating stress tolerance (Zulfiqar et al. 2021; Yang et al. 2022a, b; Zhang et al. 2022a, b; Kerbler et al. 2023; Sarkar and Sadhukhan 2023; Islam et al. 2025; Eh et al. 2025; Ibrahim 2025). Trehalose is thus a component that fulfills diverse functions ranging from a carbon or energy source (when glucose is released upon hydrolysis), signaling molecule, component of cell wall glycolipids, to, most importantly, a protective agent against various environmental stresses. Recently, the trehalose metabolic pathway has also been shown to play a crucial role as a regulator of carbon and nitrogen metabolism, as well as source-sink relationships, thereby largely influencing plant growth and development (Kerbler et al. 2023). Additionally, under stressful conditions, trehalose has been shown to have an excellent capacity to protect cellular structures, outcompeting other sugars (Delorge et al. 2014; Mohsin et al. 2022). As such, exogenous trehalose application (Abdallah et al. 2020; Abid and Shahbaz 2022; Mohsin et al. 2022; Eh et al. 2025; Islam et al. 2025) and transformation of genes implicated in the trehalose metabolic pathway, resulting in elevated salinity tolerance and high-yielding crop plants (Joshi et al. 2020; Kumar et al. 2022; Sarkar and Sadhukhan 2023; Eh et al. 2025; Islam et al. 2025). Similarly, exogenous trehalose effectively reduces heat damage at high temperatures and improves the heat tolerance of rice varieties (Xu et al. 2025). It achieves this by enhancing photosynthetic and carbohydrate metabolism, increasing antioxidant enzyme activity, raising osmoregulatory substance levels, and decreasing membrane lipid peroxidation in the flag leaves. Also, significant alterations in gene expression related to trehalose metabolism have been reported in response to a variety of abiotic stresses (Kerbler et al. 2023; Mohanan et al. 2023; Eh et al. 2025). In addition to trehalose, evidence suggests T6P (a metabolite or precursor in the trehalose biosynthesis pathway) as a metabolic signal regulating sugar and energy homeostasis, and hence is a crucial regulatory component of plant metabolism and growth under stress (Mohsin et al. 2022; Kerbler et al. 2023; Sarkar and Sadhukhan 2023; Eh et al. 2025). We therefore believe that the external application of trehalose, engineering trehalose metabolism, and an intermediate and/or an enzyme of its biosynthetic pathway are potential biotechnological markers for alleviating salinity damage and enhancing crop tolerance and productivity. Furthermore, trehalose is also present in various organisms, including bacteria, yeast, fungi, insects, invertebrates, and plants, serving a wide range of functions (Maicas et al. 2016; Tang et al. 2018; Han et al. 2024). In planktonic crustaceans, salinity-tolerant animals exhibited a positive correlation with trehalose concentrations, supporting the role of trehalose in salinity stress tolerance (Santos et al. 2024).

As for trehalose transport in plants, sugar transporters (i.e., sucrose transporters, SUT/SUC; sugars will eventually be exported transporters, SWEETs; monosaccharide transporters, MSTs) have been suggested to play essential roles in trehalose transport and distribution throughout the plants (Cha-um et al. 2019; Feng et al. 2019; Keller and Neuhaus 2025). These sugar transporters are typically categorized into two groups (Cha-um et al. 2019): (a) Those energized by electrochemical potential gradients created by proton pumps across the cellular membranes, i.e., secondary active transporters, and (b) Sugar transporters that facilitate sugars to pass across membranes down the concentration gradients, i.e., facilitated diffusion. Despite the proposed reliance of trehalose on sugar transporters for its transport and distribution in plants, a dedicated trehalose transporter remains unconfirmed in plants and is an active research area. The purpose of this review is, therefore, to deliver updated evidence that trehalose is not only a sugar that acts as a nonharmful osmoprotectant osmolyte, but also to answer the question of whether trehalose or its biosynthesis pathway intermediate (T6P) or enzyme (TPS, TPP) can be utilized as a biomarker to develop crop salinity-tolerant and yielding under saline stress. Reassessing the significance of trehalose or its metabolites in the homeostasis of crop plants that produce it under saline environments is largely important. Therefore, the review discusses the structure, biosynthesis, functional molecular mechanisms, as well as engineering and external applications of trehalose as efficient strategies to improve crop salinity tolerance and yield. Additionally, the review emphasizes trehalose and its metabolites as multifunctional bio-stimulants (cell hydration agent, antioxidant, gene regulator, source of energy and carbon, precursor for metabolic pathways, signaling molecule, and detoxifier of excess ROS) for application in sustainable agriculture to enhance crop productivity under saline conditions.

## Trehalose structure, biosynthesis, and protective molecular mechanisms

### Structure and properties

**Fig. 2:**
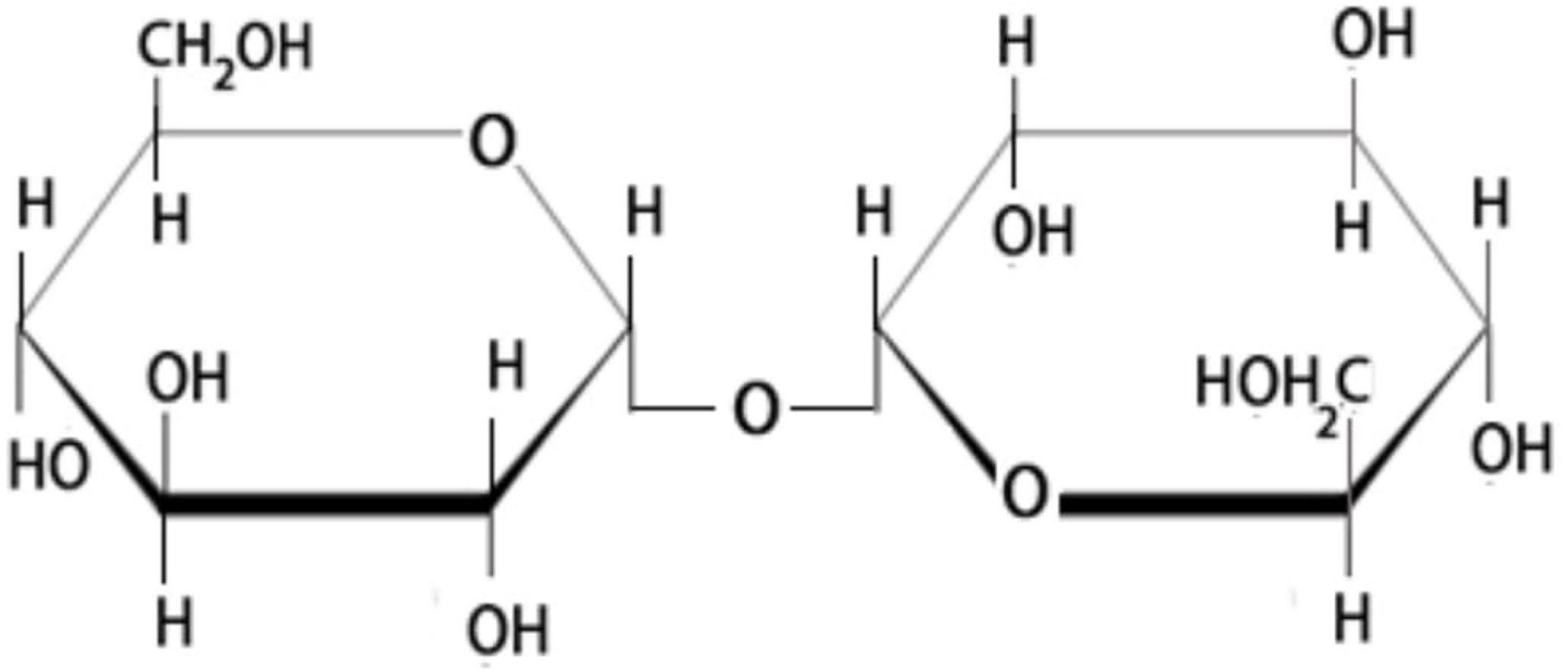
Structure of trehalose: composed of two glucose units connected by 1,1 α-glycosidic bonds. Trehalose has superior physicochemical properties that make it an exceptional sugar, as detailed in the text

Trehalose is a natural soluble nonreducing disaccharide formed by a 1,1-glycosidic bond between two α-glucose units (Fig. 2), giving it the name α-D-glucopyranosyl-(1→1)-α-D-glucopyranoside (Iturriaga et al. 2009; John et al. 2017; Han et al. 2024). This bonding keeps trehalose in closed-ring form, stabilizes its structure at high temperatures, and is very resistant to acidic conditions and hydrolysis (Figueroa and Lunn 2016; Han et al. 2024). Trehalose is also stable at a wide range of pH values. In addition, the glycosidic bond connecting the two glucose units has low energy (1 kcal/mol), resulting in a very stable structure relative to sucrose, which needs a high-energy bond (27 kcal/mol). Also, breaking trehalose into its two hexoses is not easy in the absence of trehalase enzyme (Iturriaga et al. 2009). These superior trehalose physicochemical properties make it an exceptional sugar. Further, trehalose in aqueous solutions tends to form a concentration-dependent clustering and hydrogen bonds among them, thus forming clusters of various sizes (Elbein et al. 2003). Owing to trehalose’s ability to retain water, it is utilized in food, cosmetics, and as a drug. Among the three possible anomers of trehalose: α,β-1,1-, β,β-1,1-, and α,α1,1-, only α,α-trehalose (Fig. 2) has been observed in living organisms (Elbein et al. 2003).

### Biosynthesis and hydrolysis

**Fig. 3:**
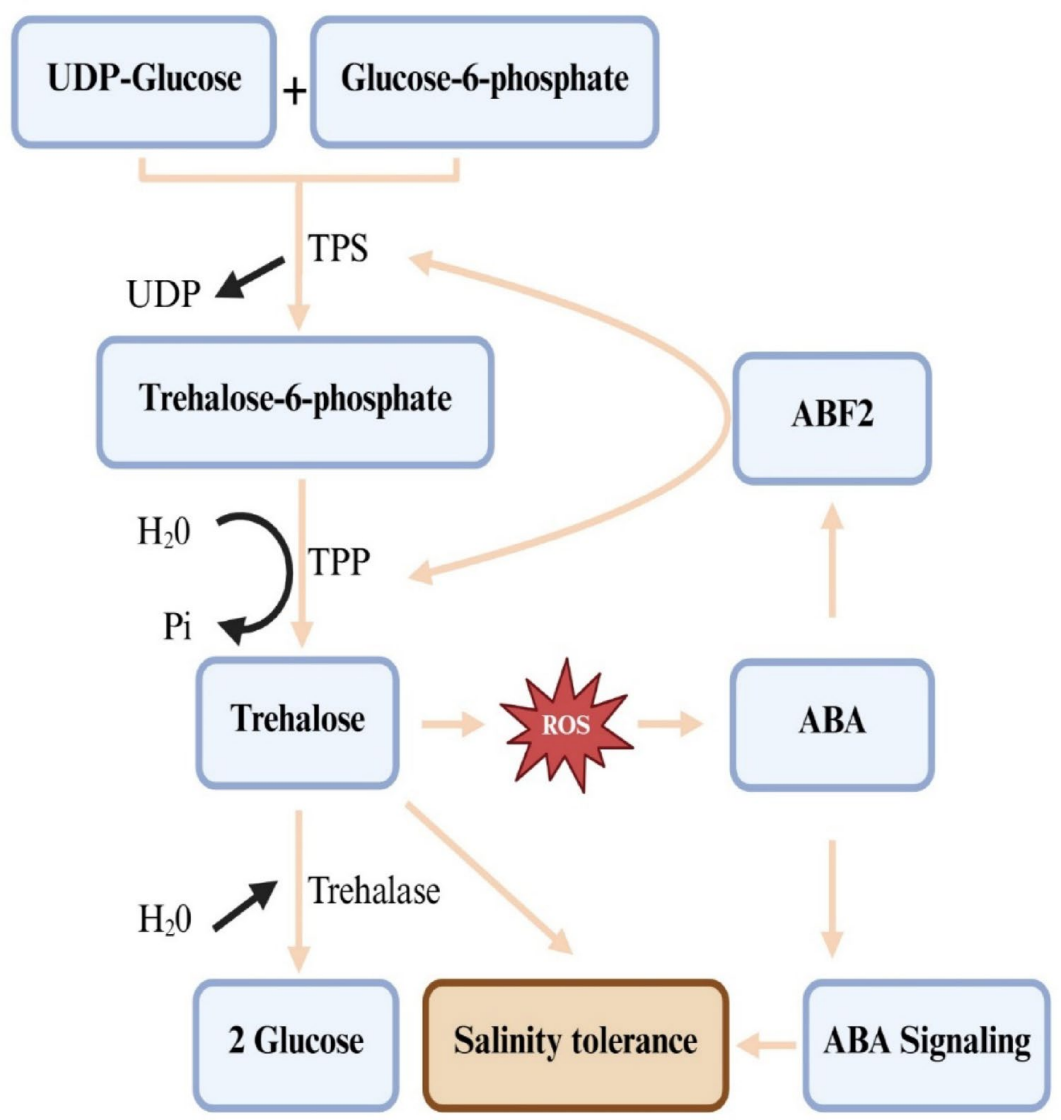
Trehalose biosynthesis and hydrolysis pathway, and the implication of ABA and ROS as salinity signals contribute to salinity stress tolerance via trehalose biosynthesis enhancement. UDP, uridine diphosphate

It was not until 1998 that genes encoding catalytically active TPS (EC 2.4.1.15) and TPP (EC 3.1.3.12) were identified in *Arabidopsis*, and the importance of trehalose metabolism led to its biosynthesis and heterologous expression of microbial *TPS* and *TPP* genes (Grennan 2007; Figueroa and Lunn 2016). These genes are identified in all major plant taxa, indicating their capacity to synthesize trehalose and demonstrating its universal presence in the plant kingdom (Kerbler et al. 2023; Han et al. 2024; Eh et al. 2025). Five biological pathways support trehalose biosynthesis in various living groups. The most common pathway that plants use consists of two steps (John et al. 2017; Eh et al. 2025): in the first step, the TPS enzyme catalyzes glucose transformation from uridine diphosphate-glucose (UDP-glucose) into glucose-6-phosphate (G6P), resulting in T6P and UDP (Fig. 3). In the second step, the TPP enzyme mediates the dephosphorylation of T6P into trehalose. TPP and T6P have been reported to be located in the chloroplasts as well as other various cellular compartments, including vacuoles, cytosols, and nuclei (Kerbler et al. 2023; Raza et al. 2024). This biosynthetic pathway is the most widely distributed in all organisms, where TPS and TPP are the major enzymes in trehalose metabolism, and also various isoforms of each are found in various plants (Delorge et al. 2014; Raza et al. 2024). For instance, 11 *TPS* genes and 10 *TPP* genes were identified in *Arabidopsis* (Delorge et al. 2014), whereas 14 *TPS* and 13 *TPP* genes were identified in rice (Han et al. 2024). The *TPS* genes are composed of class I and class II genes, where only *AtTPS1* from the four genes encodes for an active synthase in Arabidopsis. On the other hand, it is important to note that class II genes do not encode active enzymes (Delorge et al. 2014), and therefore, we suggest that future research should investigate a potential role for their proteins under stress conditions. The two genes (*TPS* and *TPP*) and their homologs, coding trehalose, were identified in different plant species, and their expression in several crop species resulted in trehalose accumulation and enhanced abiotic stress tolerance (Nawaz et al. 2022; Sarkar and Sadhukhan 2022; Hassan et al. 2023; Eh et al. 2025). However, several options should be considered to achieve more success by altering the trehalose metabolism of plants because overexpression of the trehalose biosynthetic genes leads to various growth aberrations (Delorge et al. 2014; Figueroa and Lunn 2016; Phan and Van Dijck 2019). That is, using the plant’s endogenous genes and the expression of *TPS* and *TPP* should be limited to certain levels, and at the same time, utilize stress-inducible promoters to regulate the introduced genes (Figueroa and Lunn 2016). Further, Eh et al. (2025) presented evidence that these aberrant phenotypes are related to changes in T6P levels, and the co-expression of *TPS* and *TPP* is a successful method that results in no phenotypic abnormalities. The authors report that the absence of these aberrant phenotypes in response to the co-expression of microbial *TPS* and *TPP* genes is attributed to the contention that the fusion enzymes immediately convert T6P to trehalose without accumulating the intermediate T6P. Delorge et al. (2014) also suggest modification of trehalose metabolism to improve stress tolerance without adverse effects through trehalase overexpression, which hydrolyzes trehalose into two glucose units (Fig. 3). It is worth mentioning that salinity stress-related signaling molecules such as ABA and ROS regulate *TPS* and *TPP* genes and, hence, trehalose production (Wang et al. 2020a; Song et al. 2024; Ibrahim 2025). ABA activates transcription factors like ABA-RESPONSIVE ELEMENT BINDING FACTOR 2 (ABF2) to bind to *TPS*/*TPP* promoters, which activate their expression, thus boosting trehalose production and enhancing plant tolerance to saline conditions (Fig. 3). Trehalose accumulation triggers the production of ROS, creating a feedback loop that further enhances the ABA signaling pathway and strengthens ABA’s effects on processes like stomatal closure and other stress responses, which improves salinity tolerance (Wang et al. 2020a; Song et al. 2024). For example, *AtTPPD* overexpression lines showed improved salinity tolerance due to hypersensitivity to redox changes in two cysteine residues of *TPPD*, which led to trehalose accumulation (Krasensky et al. 2014). However, it is worth mentioning that trehalose accumulation elevates ROS formation, and a signaling feedback loop that enhances the ABA signaling pathway lacks clear direct evidence and unresolved mechanisms, requiring further exploration and confirmation.

Trehalose can only be split into its monosaccharide residues under extreme hydrolytic agents or in the presence of the enzyme trehalase, which hydrolyzes trehalose into two glucose moieties (Delorge et al. 2014). It is proposed that the ubiquitous presence of the enzyme trehalase in plants may result in low amounts of trehalose, which is frequently found in various plant species (Delorge et al. 2014). Trehalose breakdown can also occur by trehalose phosphorylase, producing glucose and glucose-1-phosphate (Raza et al. 2024). Due to this controversy, it appears that trehalose itself may not be the effector, but rather an intermediate and/or an enzyme in its biosynthetic pathway that plays a crucial role in crop growth, development, and stress tolerance (Avonce et al. 2006; Grennan 2007).

### Molecular mechanisms of trehalose/T6P’s protective role

When plants are faced with stress, trehalose gradually accumulates inside the cytoplasm as a direct response, preventing cellular macromolecules from denaturing and also suppressing their aggregation. Trehalose’s protective potential has been reported to be due to mechanisms that are a result of its physicochemical properties, particularly trehalose as a non-reducing sugar (Nawaz et al. 2022; Han et al. 2024). Due to its nonreducing nature, trehalose is less chemically reactive, making it compatible with cellular metabolism even at high concentrations. Its high levels do not harm cellular macromolecules but can help preserve them (Nawaz et al. 2022). Three possible mechanisms have been suggested to explain how trehalose does not damage cellular structures: water replacement, glass formation, and chemical stability (Iturriaga et al. 2009). These trehalose protective mechanisms may all work together to stabilize the cellular macromolecules, and they are not mutually exclusive. All biological macromolecules are normally stabilized by a hydration layer around them, which is formed by hydrogen bonds between water and the cellular macromolecules. The water replacement theory suggests that trehalose preserves biological structures against dehydration, caused by different stresses, as trehalose replaces water molecules in the hydration layer. The replacement role helps to stabilize biomolecules and inhibit their irreversible denaturation (Iturriaga et al. 2009; Nawaz et al. 2022; Islam et al. 2025). This feature of trehalose is due to the flexible nature of the α-(1→1) glycosidic bond that is greater than that of the glycosidic bond of other disaccharides; this allows trehalose to easily join the polar groups of the molecules and hence to have compatible functions with them (Paul and Paul 2014). Additionally, other disaccharides can displace water, but the trehalose hydration number exceeds that of the other disaccharides (Iturriaga et al. 2009). In this respect, Bezrukavnikov et al. (2014) report that DNA bases have a higher affinity for trehalose than water, which promotes the melting of double-stranded DNA and stabilizes single-stranded nucleic acids. Secondly, trehalose can crystallize into a glass-like appearance under extreme dehydration; a particular property of trehalose that preserves cellular macromolecules from denaturation and recovers their functional activity upon rehydration (Kosar et al. 2019). Another function of this glassy formation is its ability to limit molecular motion and prevent protein aggregation and free radical diffusion under desiccation stress (Delorge et al. 2014; Figueroa and Lunn 2016). The previous finding indicates that the key difference that makes trehalose surpass other disaccharides is its ability to form this glassy structure around molecules even in the absence of water, ensuring their stability even under harsh conditions (Paul and Paul 2014; Figueroa and Lunn 2016). It is worth mentioning that this glassy state has also been reported in low concentrations of trehalose, which was attributed to the transition from weak to strong correlated hydrogen bonds (Olgenblum et al. 2020). Thirdly, the chemical stability of trehalose is attributed to the strong resistance of the α-(1→1) glycosidic bond to cleavage by glucosidases and acid hydrolysis, which is why trehalose remains stable in solution at high temperatures and acidic conditions (Crowe et al. 1984). As mentioned earlier, this bonding of trehalose has low energy (1 kcal/mol) compared with another similar disaccharide, sucrose (27 kcal/mol). Because of these peculiar molecular features of trehalose, a focus of interest on its role in improving the performance of crop plants under various abiotic challenges has been reported (Abdallah et al. 2020; Lu et al. 2021; Yang et al. 2022a, b; Liu et al. 2022; Kerbler et al. 2023). The previous works report that trehalose efficiently stabilizes dehydrated enzymes, proteins, biological structures, and membrane lipids. This helps to preserve their structures and hence protects them against the harsh impact of various abiotic stresses. Additionally, trehalose enhances crop salinity tolerance by mitigating the adverse effects of oxidative stress. It acts through the activation of the antioxidant defense system and/or as a ROS scavenger, thereby lowering ROS generation and their detrimental effects (Islam et al. 2025). However, the latter role of trehalose as a ROS scavenger has not been sufficiently confirmed and needs further investigation and documentation. The signaling metabolite T6P, on the other hand, has the potential to relate reducing power to energy metabolism, thereby fueling growth, as detailed in a forthcoming section.

Regarding the molecular mechanisms linking trehalose and T6P to salinity stress tolerance, T6P has been shown to serve as a crucial signal connecting carbohydrate metabolism to growth and stress responses (Sarkar and Sadhukhan 2022; Eh et al. 2025; Ibrahim 2025; Waqas et al. 2026). That is, T6P is a central metabolic regulator that links carbohydrate availability to growth and stress responses. Moreover, Elevated T6P levels promote growth pathways, while lower T6P levels trigger the mobilization of carbon reserves for stress response. T6P can also regulate the expression of genes involved in stress tolerance and thus modulate plant resistance to salinity (Waqas et al. 2026). On the other hand, trehalose has been reported to act as a compatible solute and a direct osmoprotectant to maintain water potential and prevent cellular damage, stabilizing cell membranes and proteins, and upregulating antioxidant genes (Nawaz et al. 2022; Yang et al. 2022a, b; Eh et al. 2025; Ibrahim 2025). In short, T6P acts as a sensor and signaling molecule, modulating cellular resources towards survival under salinity stress, while trehalose directly mitigates the damaging effects of salinity on cellular structures and functions.

## Functions of trehalose and its metabolites

In response to salinity stress impacts, crop plants employ a wide range of tolerance mechanisms; some are constitutive, while others are activated only when specific stress signals are detected (John et al. 2017). These include modifying signal transduction pathways, boosting downstream gene expression, maintaining ion homeostasis, and accumulating harmless osmolytes in response to stress (Figueroa and Lunn 2016; Nawaz et al. 2022; Younis and Mansour 2023). Among compatible osmolytes, trehalose and T6P are especially important, serving multiple functions involved in adaptive responses and stress tolerance of crop plants (Sarkar and Sadhukhan 2022; Zhang et al. 2022a, b; Sharma et al. 2023; Eh et al. 2025; Waqas et al. 2026). It is important to note that once stress conditions are alleviated, trehalose levels return to normal, reflecting its crucial role during stress. However, the functions of trehalose in plants remain debatable because transgenic plants overexpressing trehalose biosynthetic genes often show a variety of phenotypic abnormalities and stress symptoms (Delorge et al. 2014; Yang et al. 2014; Kosar et al. 2019; Bánfalvi 2019). These challenges can be addressed by modifying endogenous genes responsible for trehalose synthesis, with careful consideration of subcellular locations, specific cell types, tissue categories, and developmental stages. Targeted approaches could also reduce growth-related issues linked to trehalose. Eh et al. (2025) suggest that overexpressing microbial *TPS* and *TPP* fusion genes may be a promising strategy to avoid abnormal phenotypes. This evidence suggests that plants have safeguards to maintain their machinery under adverse conditions, and to do that, they must incur some loss, to some extent. Furthermore, evidence also indicates that trehalose itself might not be the direct effector but rather an intermediate (T6P) or an enzyme involved in its synthesis (TPS, TPP), which play pivotal roles in plants under stress (Nawaz et al. 2022; Hassan et al. 2023; Waqas et al. 2026). This section discusses and evaluates the various roles of trehalose or its metabolites in enhancing plant tolerance to salinity stress. These mechanisms likely all contribute to the protective functions of trehalose and are not mutually exclusive.

### Regulation of crop growth, development, and carbohydrate metabolism

In response to saline conditions, plant content of trehalose, its metabolic intermediate, or an enzyme involved in its biosynthesis plays a key role in the regulation of growth, development, and metabolism (Wang et al. 2020a; Onwe et al. 2022; Hassan et al. 2023; Islam et al. 2025). One crucial role of trehalose, as reported by Yang et al. (2014), is its impact on plant vegetative and floral growth under salinity stress. In the work of Yang et al., both vegetative and reproductive growth were largely improved by trehalose supply, highlighting the possible role of trehalose in helping plants to antagonize salinity stress in part by the completion of the life cycle under saline conditions. It is most likely that the role of trehalose during plant salinity response is through its importance for crop productivity, as severe salinity stress will drastically interrupt plant floral growth, which eventually affects yield potential. Trehalose treatment also increased the growth parameters and yield of various plants, which declined under saline soil; quinoa plants (Abdallah et al. 2020), wheat cultivars (Fordil and Khan 2022), and *Catharanthus roseus* (Chang et al. 2014). The improved growth and resistance to high salinity observed in the above works by trehalose were always associated with ionic regulation, osmotic adjustment, and enhanced antioxidant defense system. In the same trend, trehalose metabolism disruption was associated with severe alterations in embryogenesis, flower formation, organogenesis, growth, and senescence, highlighting trehalose and T6P essential roles in plant growth and development at all growth stages (Ponnu et al. 2011; Kerbler et al. 2023; Waqas et al. 2026). In addition, trehalose impacts sugar metabolism because the reduced level of trehalose triggered by trehalase hydrolysis of trehalose causes a drastic decline in starch and sucrose content (Nawaz et al. 2022), suggestive of trehalose’s role in carbon allocation and sugar metabolism. Also, T6P, which is an intermediate in trehalose biosynthesis, is crucial for embryonic and vegetative development of plants, and sucrose and starch metabolism (Onwe et al. 2022; Kerbler et al. 2023). The major role of T6P is to signal and regulate sucrose levels under normal and stressed conditions via acting as a negative feedback regulator (Ponnu et al. 2011; Kerbler et al. 2023), although how T6P senses and regulates sucrose levels remains unclear and requires further exploration. However, Blanford et al. (2024) and Waqas et al. (2026) recently reported that T6P is a key for cellular sugar status and a strong inhibitor of Sucrose non-fermenting 1-related protein kinases 1 (SnRK1) through binding to SnRK1 catalytic subunit (KIN10), which disrupts the activation loop and precludes its phosphorylation and activation by GRIK1 under high sugar. Under these conditions, SnRK1 minimally phosphorylates its target proteins, switching from catabolism to anabolism (Blanford et al. 2024). In this respect. Eh et al. (2025) also indicate that when the T6P level is low, it enhances SnRK1 activity and cellular metabolism shifts toward the famine direction, strengthening stress responses. Conversely, when the T6P level is high, SnRK1 activity is suppressed, causing plant cell metabolism to shift towards a feast state, which in turn increases biomass and yield. Other studies report that trehalose regulates embryo maturation, plant growth and development, flowering, carbohydrate and abscisic acid metabolism, as well as stress signaling (Iturriaga et al. 2009; Sah et al. 2016). In this respect, it is worth noting that the growth abnormalities observed in the transformed plants are possibly due to the accumulation of T6P, confirming that T6P is a key mediator in plant development. Our proposal is supported by the finding that homozygous *tps1* mutants exhibited embryo-lethal phenotypes in *Arabidopsis*. Similarly, when the *TPS* gene was rendered non-functional, aquatic invertebrate genotypes could not produce trehalose and suffered drastically decreased salinity tolerance, emphasizing the importance of this sugar and its metabolite in their survival (Santos et al. 2024). As such, the *AtTPS1* gene has been reported to be essential for root and shoot growth as well as for the transition to flowering (Hassan et al. 2023). Furthermore, T6P has been reported to inhibit SnRK1, which is an important transcriptional regulator of metabolism, growth, and development in plants (Iordachescu and Imai 2011; Kerbler et al. 2023). As detailed above, T6P obviously acts as a potent switch for plant growth, carbon status, and catabolism under normal and stressful conditions (Blanford et al. 2024; Waqas et al. 2026). Phan and Van Dijck (2019) also report that the role of T6P as a signaling molecule, a regulator of plant metabolism, growth, and development, becomes clear because trehalose-induced growth inhibition is related to T6P accumulation in Arabidopsis seedlings. High trehalose levels might lead to T6P dephosphorylation reduction, increasing T6P content. In this work, the growth inhibition by T6P was restored by adding metabolizable sugars to the growth medium enriched with trehalose. This finding reveals that when the balance between T6P contents and sugar availability is impaired, growth is reduced, which can be restored by the addition of sucrose to the medium, suggesting that T6P improves plant growth when the carbon supply is high. The results suggest that trehalose metabolism is important for plant development, as T6P adequate levels are essential for carbon utilization during normal growth. Based on this, it is obvious that the growth and reproduction of the salinity-stressed crop plants largely benefit from trehalose and/or its biosynthesis intermediates and enzymes.

Another example supporting the important role of T6P in regulating crop metabolism in response to salinity imposition is the sucrose: T6P ratio (Henry et al. 2015; Kahraman et al. 2019; Kerbler et al. 2023; Waqas et al. 2026). In plant cells, the sucrose: T6P ratio has been reported to affect various metabolic processes through the induction or repression of transcription factors in response to different stresses. For instance, elevated T6P levels repress the catalytic activity of SnRK1, a key transcriptional regulator affected by carbon and energy availability (Iturriaga et al. 2009; Nuccio et al. 2015; Waqas et al. 2026). Consequently, T6P exhibits an adverse effect on genes that are upregulated by SnRK1 while simultaneously exerting a beneficial influence on genes that are downregulated by SnRK1 (Kahraman et al. 2019). Furthermore, T6P appears to function as a negative feedback regulator, modulating sucrose concentrations through its interaction with SnRK1, which was previously discussed (Blanford et al. 2024). This regulatory loop between T6P and SnRK1 leads to a balance in growth when energy status is favorable, or restrains metabolism when energy status is low. Henry et al. (2015) further reported that the interaction between T6P and sucrose is a developmental stage-, tissue-, and cell-type-dependent process, and is regulated by environmental factors. Proposed mechanisms for the inhibition of SnRK1 by T6P are forwarded by Kerbler et al. (2023); T6P inhibits SnRK1 via interaction with unknown protein factors and indirectly by inhibiting the interaction of SnRK1 with SnRK1 activating kinases (SnAKs GRIKs). They also indicate that class II TPS proteins and non-catalytic TPS-like proteins similarly act as inhibitors of SnRK1. This suggestion, presented by Kerbler et al. (2023), is currently further unmasked by the work of Blanford et al. (2024). It, therefore, appears that T6P and SnRK1 signaling are involved in stress response and recovery by regulating genes that utilize sucrose in growth and development, as well as the synthesis of end products. The evidence indicates that metabolism of trehalose, via its intermediates and associated enzymes, is crucial in regulating the distribution of carbon and the coordination between sources and sinks. Another transcription factor that regulates carbohydrate metabolism and is controlled by T6P is basic leucine zipper 11 (bZIP11) (Henry et al. 2015). Therefore, it can be concluded that T6P indirectly modulates the developmental phase transitions, carbohydrate, and amino acid metabolisms, as these processes are regulated by bZIPs. This contention is supported by the finding that when trehalose is supplied in high concentrations, the observed plant toxicity has been linked to T6P accumulation and bZIP11, as its overexpressing plants show insensitivity toward supplied trehalose (Delatte et al. 2011). It appears that a connection between T6P, SnRK1, or bZIP11 possibly explains the resulting toxicity of trehalose, and most likely impacts responses and tolerance to stress. It is, therefore, reported that manipulating T6P levels through genetic modification or chemical intervention is a powerful tool to enhance crop production (Waqas et al. 2026). We recommend that, in engineering interventions, T6P should be precisely modified and targeted to specific cell types and tissues to avoid unfavorable responses, which should be considered in future research dealing with various exogenous trehalose concentrations.

A further feature of trehalose’s impact on plant growth and development is provided by the fact that trehalose and ABA have synergistic effects on root growth and stomatal closure (Wang et al. 2020a). In this respect, ABA has been found to stimulate trehalose accumulation, and trehalose external supply significantly enhanced ABA biosynthesis in both the roots and leaves of *Avicenna marina* (Song et al. 2024). The expression level of *TPPE*, as one of the ten genes encoding TPPs and trehalose levels in *Arabidopsis* increased in response to ABA: the ABA-responsive transcription factor ABA responsive element-binding factor2 (ABF2) in the presence of ABA directly binds to the *TPPE* promoter and activates its expression. *TPPE* is, therefore, implicated in ABA‐controlled stomatal movement and root elongation, since in the *tppe* mutant, stomatal closure was less sensitive to ABA than wild-type plants (Kerbler et al. 2023). Similarly, Song et al. (2024) have confirmed the direct binding of AmABF2 to the *AmTPS9A* promoter, activating its expression and activity in vitro and in vivo. Accordingly, the results uncover a new aspect of the ABA signaling pathway and provide a molecular basis for the trehalose role in crop responses to abiotic stresses. Moreover, it has recently been demonstrated that trehalose metabolic pathways are also important for normal seed germination (Huang et al. 2023). For instance, hydrogen gas-enhanced seed germination in cucumber was achieved by enhancing trehalose biosynthetic enzyme activity and gene expression, decreasing starch level through promoting related enzyme activities and their gene expression, thereby elevating the endogenous trehalose level (Huang et al. 2023). It is, therefore, evident that hydrogen crosstalks with trehalose serves as a signaling molecule that plays an important role in seed germination. In conclusion, the regulation of plant growth, development, and sugar metabolism by T6P/trehalose is, therefore, a noncontroversial fact. Their precise modulation is, therefore, recommended for biotechnological interventions to enhance crop resilience and yield potential, which surely contributes to sustainable agriculture and global food security.

### Trehalose/T6P induces other high sugar levels, serving as an energy and carbon source

Sugars have been demonstrated to provide carbon and energy for normal cellular metabolism and regulate plant metabolism, growth, and development under normal and stressed conditions (Sharma et al. 2023). In support, salinity-induced starch accumulation in tomato roots and leaves reduced the photosynthetic performance, chlorophyll content, and starch transport. At the same time, exogenous trehalose treatment overcomes these adverse effects and supplies the necessary sugars and energy for resilience processes (Feng et al. 2019). Also, trehalose accumulation has been shown to influence sugar metabolism that is required for the osmoprotectant role under various environmental stresses (Redillas et al. 2012; Delorge et al. 2014; Yasseen et al. 2018; Islam et al. 2025). Research by Sah et al. (2016) similarly showed that nonreducing disaccharides (trehalose and sucrose) provided a soluble energy source as stable molecules, as well as acting as a protectant compound in crop plants in response to stress conditions. The previous authors reported that accumulated trehalose served as a storage carbohydrate that possesses the unique feature of reversible water absorption capacity to maintain cell turgidity and protect biological molecules from stress-induced dehydration. In this respect, trehalose is superior to other sugars, conferring protection. In a study by Abdallah et al. (2016), trehalose priming induced the accumulation of soluble sugar when rice cultivars were subjected to saline conditions, suggesting trehalose’s crucial role as a modulator of other carbohydrate molecules and sugar transport. Additionally, trehalose supply to salinity-stressed *Arabidopsis* resulted in improved shoot fresh weight and leaf water content relative to untreated plants. The response was explained by the fact that trehalose induced the enzymes that catalyze the accumulation of storage carbohydrates in photosynthetic tissues (Yang et al. 2014). Similarly, exogenous trehalose increases the activities of sucrose synthetic enzymes (sucrose phosphate synthase and sucrose synthase) and soluble sugars (trehalose, sucrose, glucose 6-phosphate, fructose 6-phosphate), which improves the energy status and membrane stability, and lowers chilling adversities in guava fruit stored in a cold environment (Vichaiya et al. 2022). Another recent work by Vichaiya et al. (2023) illustrated that postharvest trehalose supply enhanced the expression of energy-producing related genes that encode (i.e., adenosine triphosphate synthase subunit β, adenosine diphosphate/adenosine triphosphate carrier) and stimulated the enzyme activities of nicotinamide adenine dinucleotide dehydrogenase, succinate dehydrogenase, and cytochrome c oxidase. These trehalose-inducible responses were associated with higher energy charges and reduced chilling damage in cold storage guava fruits. These findings suggest that trehalose supply results in a transient increase in sucrose levels at early storage to act as sugar signaling to SnRK1 activation; this is required for maintaining energy homeostasis needed for chilling injury suppression. In a field experiment, a foliar spray of trehalose improved yield, yield components, and chemical composition of quinoa seeds under drought stress, which was attributed to trehalose serving as a storage carbohydrate and transport sugar (Tarek et al. 2017). Interestingly, Raza et al. (2024) reported that T6P increased maize growth and development by improving carbon availability under cold stress. Also, as T6P has a dual function (a signal molecule and homeostatic regulator of sucrose levels), Kerbler et al. (2023) indicated that T6P in source leaves controls the sucrose production to balance supply with demand for sucrose from growing sink organs. Moreover, in addition to T6P role as an important signaling metabolite, T6P regulates carbon assimilation and sugar status in plants and plays an essential role in plant development (Ponnu et al. 2011; Waqas et al. 2026). Based on that, trehalose/T6P has been demonstrated to indirectly have important roles as a carbon storage, energy source, and carbon transport molecule by triggering the synthesis of high levels of other sugars in response to stress conditions. However, Kerbler et al. (2023) argued that a low level of trehalose, always found in higher plants, is inconsistent with its role as carbon storage. We therefore propose that other high levels of sugars regulated by trehalose may serve as carbon sources for energy, growth, and maintenance purposes in response to stress.

Further evidence concerning indirect trehalose’s role as an energy and carbon reserve is provided in this section. For example, one crucial role of trehalose in inducing plant-rhizobia interaction and thus improving abiotic stresses is its role in acting as a source of carbon and energy in different phases of the rhizobium-legume association (Bharti et al. 2022). Feng et al. (2019) also demonstrated that an external supply of trehalose significantly upregulates the expression of fructokinase and phosphofructokinase in tomato plants under salinity stress, suggesting trehalose’s capacity to enable plants to accumulate sugars and maintain efficient respiration. The work also illustrated that, in addition to sugar’s role as the primary energy source for plant growth, it can also serve as a signal molecule under stress, as trehalose treatment upregulates sugar signal transduction genes in tomato under salinity stress. Furthermore, the expression level of the monosaccharide transporter (*MST3*) gene and the sucrose transporter genes *SUT1* and *SUT4* were significantly upregulated under salinity and trehalose treatment (Feng et al. 2019). The results suggest trehalose’s ability to alter the source-sink transport pathway of sugars, thereby regulating the accumulation and distribution of other sugars to provide the required source of carbon and energy for tomato plants. It appears that tomato plants were unable to utilize carbon sources under salinity stress. Sadak (2019) similarly revealed that trehalose foliar application enhanced growth parameters and accumulation of glucose, sucrose, trehalose, starch, and soluble sugars of salinity-stressed plants, indicative of the indirect impact of trehalose on carbohydrate metabolism to serve in turgor maintenance and as a reserve form of carbon. Under drought conditions, Bao et al. (2024) reported that foliar spray of trehalose or T6P promoted starch, soluble sugar, and lignin accumulation in the petals, pointing to the role of T6P or trehalose as a positive regulatory signal participating in the accumulation of various sugars and enhancing the drought tolerance of rose plants. In summary, although trehalose (a soluble, non-reducing disaccharide) is present in low amounts in plants, it modulates sugar accumulation and distribution by affecting the activities of sugar transporters and simultaneously regulating sugar metabolism to adequately supply carbon and energy sources, thereby significantly improving plant salinity stress resistance. As trehalose is not always present in high enough levels in plants, it is obvious that trehalose’s role is mainly to modulate other sugar accumulation in sufficient amounts to satisfy the required energy and carbon sources under stress.

### Induction of the expression of stress-responsive genes

**Fig. 4:**
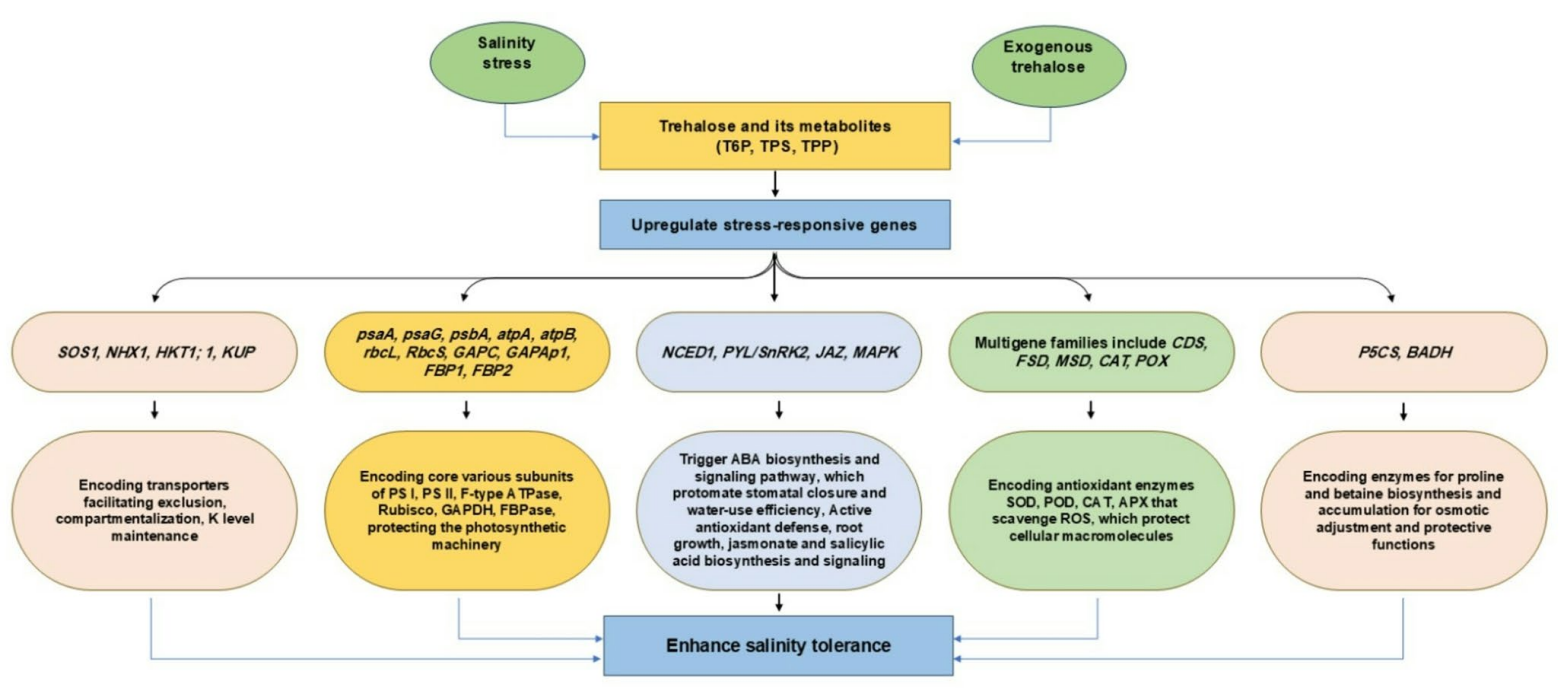
Salinity-induced and exogenous trehalose regulate stress-responsive genes that encode enzymes and proteins, thereby modulating ion homeostasis, osmolyte biosynthesis and accumulation, antioxidant defense systems, crosstalk with hormone signaling, and protection of photosynthetic machinery. Collectively, enhance tolerance to saline conditions

Induction of stress-responsive genes by trehalose or its metabolite T6P, which increases tolerance to salinity stress, is summarized in Fig. 4. Several reports indicate that trehalose-induced upregulation of stress-responsive genes enhances crop production and resilience to stress conditions (Nawaz et al. 2022; Kerbler et al. 2023; Han et al. 2024; Waqas et al. 2026). Products of these genes participate in tolerance traits in response to stress. For example, Feng et al. (2019) analyzed metabolic changes in sugar and ABA in tomato seedlings treated with trehalose and salinity. They also examined the differential expression of key genes involved in sugar metabolism. The authors report that external trehalose decreased starch content and increased soluble sugars by modulating gene expression related to starch and sugar metabolism. The results also showed that exogenous trehalose alters sugar accumulation and distribution by upregulating sugar transporter genes and increasing ABA levels. This improves salinity tolerance by regulating genes involved in ABA synthesis and metabolism. Similarly, trehalose added to 2-week-old liquid cultures containing *Arabidopsis* changed the transcript levels of transcription factors, cell wall components, nitrogen metabolism, stress-responsive genes, defense-related genes, and fatty acid biosynthesis genes (Bae et al. 2005b). This suggests that trehalose or its metabolite (T6P) regulates gene expression, including *TPS* and *TPP*, which are involved in stress resilience mechanisms. Another study by Bae et al. (2005a) found that exogenous trehalose applied to *Arabidopsis* seedling cultures quickly altered disaccharide levels and induced detoxification and stress response proteins, highlighting trehalose’s role in modulating stress-inducible genes. Additionally, Yang et al. (2014) demonstrated that exogenous trehalose not only increases salinity tolerance in *Arabidopsis* but also boosts other processes, such as ion homeostasis and ROS scavenging, both of which are involved in salinity stress response. Further evidence showed that trehalose can trigger salinity-responsive genes that regulate various processes promoting salt tolerance. Jiang et al. (2014) reported that transferring the *IbTPS* gene from sweet potato into tobacco resulted in transgenic tobacco with enhanced salinity tolerance, higher trehalose and proline levels, and upregulated stress-related genes (*TPP*, *HSP70*, *DHN*) compared to wild-type plants. These findings suggest that enzymes involved in trehalose biosynthesis contribute similarly to increased salinity tolerance. Microarray analysis by Sah et al. (2016) clearly indicates that both trehalose and T6P influence the expression of genes involved in abiotic stress responses. External application of trehalose or T6P also induces drought-responsive genes under drought conditions, although gene responses vary, indicating functional differences between T6P and trehalose (Bao et al. 2024). Phan and Van Dijck (2019) similarly demonstrated that trehalose-induced abiotic stress tolerance may result from the upregulation of stress-responsive genes and transcription factors across various crops, rather than solely acting as an osmoprotective molecule. Furthermore, Kerbler et al. (2023) reported that both T6P and TPP may serve as signaling molecules, stress-inducible gene regulators, water content enhancers, ROS scavengers, and stomatal conductance promoters, all of which are involved in stress responses and resistance. Based on the previous evidence, it is clear that trehalose and its metabolites act as modulators of stress-responsive genes, helping detoxify the stress-harmful effects and improve overall stress tolerance.

Further investigations support the fact that the enhancement of trehalose-mediated abiotic stress tolerance is linked to the activation of stress-inducible genes and transcription factors, in addition to its role as a protectant molecule. For example, the reduced harmful impact of salinity by applying exogenous trehalose in rice was related to preserving root integrity, lowering Na^+^ accumulation, and activating genes responsible for osmotic adjustment (Kahraman et al. 2019). Additionally, a study by Li et al. (2011) revealed that in rice *TPS* overexpressing lines, trehalose and proline levels were significantly elevated whether stress was present or not, and the expression of stress-related genes such as *ELIP*, *HSP70*, *CRP*, *DHN6*, *LEA14A*, and *WS118* was increased up to twofold in transgenic plants compared to wild-type. Recently, exogenous trehalose not only increased antioxidant enzyme activities but also upregulated related gene expression (*SlCu/Zn-SOD*, *SlFe-SOD*, *SlMn-SOD*, *SlPOD*, and *SlCAT*) in salinity-stressed tomato leaves (Yang et al. 2022a), greatly helping tomato plants counteract salinity’s harmful effects. Similarly, foliar application of trehalose to rice cultivars enhanced antioxidant enzyme activities and their gene expression, resulting in reduced ROS levels under drought stress (Mohanan et al. 2023). Another study by Hathout et al. (2015) showed that trehalose-induced inhibition of the enzyme trehalase caused trehalose accumulation in tissues, which was associated with increases in starch and protein levels and the appearance of new protein patterns in two rice cultivars grown in saline soil. The results indicate trehalose role as a regulator of genes involved in mechanisms that contribute to rice salinity stress tolerance. Recent research by Vichaiya et al. (2023) demonstrated that postharvest trehalose application upregulated energy-related gene expression, resulting in higher energy levels and reduced chilling injury during cold storage of guava. Furthermore, Henry et al. (2015) reported that salinity in maize led to increased expression of several genes involved in trehalose and sucrose metabolism, along with modifications in many sugar metabolic intermediates, glycolysis, and the tricarboxylic acid cycle. A 2- to 4-fold increase in T6P levels was observed in leaves, kernels, and cobs at the silking stage. These findings suggest a broad reprogramming of gene expression and core metabolic pathways in trehalose-treated maize under salinity stress. However, Figueroa and Lunn (2016) argued that it remains unclear whether the rise in T6P during the earliest developmental stage (silking) is a primary response to salinity stress that drives other gene and metabolic changes or if the altered T6P levels and T6P/sucrose ratios reflect a broader metabolic adjustment in salinity-stressed plants. We believe both scenarios could contribute to the observed effects because both mechanisms may induce such metabolic reprogramming, and they are not mutually exclusive. Collectively, these findings report that trehalose, its precursors, and the enzymes involved in its synthesis act as regulators of genes linked to salinity stress responses and tolerance across various crops (Fig. 4). It is worth noting that although the upregulation of trehalose biosynthesis and the application of exogenous trehalose have shown protective, regulatory functions, and enhanced stress-responsive gene expression, the precise mode of action of trehalose or its metabolites in promoting these gene responses still requires further investigation.

### Upregulation of antioxidant systems to scavenge ROS

Salinity stress induces ROS production, which causes injury to macromolecules and membranes (Zhang et al. 2022b; Kesawat et al. 2023; Han et al. 2024). Therefore, the joint action of both antioxidant defense systems (i.e., enzymatic and non-enzymatic antioxidants) is vital to detoxify ROS and overcome salinity-induced oxidative stress in crop plants. Trehalose is one of the remarkable protectants due to its role that works directly to suppress the ROS (although this direct impact as an antioxidant needs more documentation) or indirectly to activate other antioxidant systems and hence stabilizes the cellular membranes, proteins, and protein complexes under abiotic stresses (Mostofa et al. 2015; Onwe et al. 2022; Raza et al. 2024; Islam et al. 2025). However, when crops are overwhelmed by stresses and the perturbation becomes more dominant or evolves from various sources, an external application of trehalose or other relevant natural secondary metabolites (e.g., choline, β-carotene, anthocyanin) could be a promising strategy to combat the hazardous effects of stress. For instance, trehalose treatment efficiently elevates the endogenous trehalose concentration, which mainly induces the antioxidant defense system by enhancing the activities of antioxidant enzymes (superoxide dismutase, catalase, peroxidase) in two rice cultivars and quinoa plants, which improve plants’ capability to scavenge excessive ROS and overcome salinity stress effects (Mostofa et al. 2015; Abdallah et al. 2016, 2020). In quinoa plants, in addition to activation of antioxidant enzymes, trehalose also stimulates nonenzymatic antioxidants (soluble sugars, trehalose, proline, free amino acids), clearly indicating trehalose’s capacity to upregulate both antioxidant defense systems, besides its direct effect as an antioxidant. Yang et al. (2014) also demonstrated that exogenous trehalose decreased ROS, which minimizes the adverse effects of salinity stress: the results indicate that trehalose alleviated salinity-induced cellular ROS and the programmed cell death (PCD) process by enhancing the antioxidant enzyme activities and the level of compatible solutes serving as nonenzymatic antioxidants, which modulate plant salinity responses and promote tolerance to high salinity. Luo et al. (2008) provided direct evidence that trehalose itself plays a key role in scavenging ROS in wheat under heat stress, indicative of trehalose’s role as an antioxidant. Additionally, overexpression of *OsTPS1* enhanced rice tolerance to multiple abiotic stresses by increasing trehalose and proline levels as well as activating abiotic stress-related genes (Li et al. 2011). The accumulation of compatible osmolytes, such as proline and glutamate, was also found to remarkably improve the performance of salinity-stressed Catharanthus roseus plants when exogenous trehalose was applied (Chang et al. 2014). It appears that elevated trehalose, proline, and glutamate act as non-enzymatic antioxidants, minimizing oxidative damage induced under saline conditions. Recent investigations have positioned trehalose as a significant factor in enhancing the salinity tolerance of wheat: this was linked to its capacity to diminish ROS levels, increase the concentration of antioxidant compounds, and promote the synthesis of compatible osmolytes when exposed to NaCl stress (Sadak 2019). The trehalose’s role in preventing salinity damage during stress was by acting as an antioxidant, triggering other antioxidants, and as a signaling molecule. Moreover, Feng et al. (2019) and Islam et al. (2025) show that trehalose enhances the antioxidant enzyme activities and increases the proline level while reducing the MDA content, therefore, minimizing the adversities caused by salinity stress in tomato and Indian mustard plants. It is worth mentioning that exogenous trehalose enhanced antioxidant enzyme activities not during salinity stress but during the recovery period in rice seedlings; the effect was more pronounced in the salinity-sensitive cultivar (Nounjan and Theerakulpisut 2012). This finding is suggestive of trehalose’s crucial role in eliminating oxidative stress in response to salinity imposition. The impact of trehalose during the recovery period is unclear, but its presence and effect may be extended after stress release in rice seedlings. Taken together, the results suggest that trehalose itself acts as an antioxidant, or by inducing the generation of non-enzymatic antioxidants and/or enhancing enzymatic antioxidant activities, enables plants to cope with salinity-induced ROS bursts and therefore contributes to the enhancement of tolerance to saline conditions. However, because of trehalose’s special chemical characteristics mentioned earlier, trehalose has been indicated to act as an antioxidant, although its underlying molecular mechanisms remain unclear, speculative, and poorly understood. This unconfirmed model, therefore, necessitates further research and exploration.

Additional evidence supporting the role of trehalose or its derivatives in boosting the antioxidant defense system under stress is provided here. For example, researchers have reported that trehalose is a key regulator of antioxidant defense systems in plants exposed to various stress conditions (Sadak 2019; Kosar et al. 2021; Zulfiqar et al. 2021; Raza et al. 2024; Bao et al. 2024; Islam et al. 2025). Under drought stress, trehalose largely improves proline content and protects the biological molecules by enhancing the antioxidant defense system in flax plants (Abid and Shahbaz 2022). Similarly, a foliar spray of trehalose or T6P elevated the production of secondary metabolites and soluble sugars that serve as non-enzymatic antioxidants under drought stress (Bao et al. 2024). In a recent study by Wang et al. (2022), trehalose-treated peaches had higher internal trehalose, sucrose, proline, and choline contents under chilling stress; these induced solutes most probably contribute to ROS scavenging and hence lower levels of chilling injury. Trehalose at 10 mM enhanced enzymatic antioxidant and glyoxalase activities, suggesting enhancement of ROS detoxification and reducing methylglyoxal in two maize genotypes under 150 mM NaCl stress (Rohman et al. 2019). Both effects protected maize genotypes against the harmful effects of salinity stress, improving genotype resistance. Rohman and coworkers’ research also revealed that trehalose enhanced a reduction in lipid peroxidation, MDA, and ROS accumulation in response to salinity imposition, altogether pointing out trehalose’s capability to enhance maize salinity tolerance through regulating other antioxidants as well as glyoxalase systems. Moreover, Zulfiqar et al. (2021) illustrated that trehalose treatment also elevated the internal content of trehalose, glycine betaine, and proline as well as the enzymatic antioxidant activities in sweet basil, a response that promotes drought tolerance. Also, increased antioxidant enzyme activities and their gene expression in leaves of rice cultivars in response to foliar trehalose application diminished ROS levels and reduced leaf electrolyte leakage under drought stress (Mohanan et al. 2023). Recently, Islam et al. (2025) reported that leaf-applied trehalose significantly increased Indian mustard salinity tolerance and yield by enhancing ion homeostasis, photosynthesis efficiency, antioxidant defense mechanisms, chlorophyll content, osmolyte accumulation, stomatal aperture, cell viability, and ROS scavenging under salinity stress. Based on the above sources, trehalose or its metabolites effectively modulate the cellular redox state, cell death, antioxidant defense system, and compatible osmolytes to control various crop performances and improve their resilience to salinity and other abiotic stresses. Therefore, we recommend trehalose as a cost-effective strategy for alleviating the salinity impacts on crop plants. However, further research is needed to explore how trehalose boosts antioxidant activities and the signaling crosstalk between this sugar and the antioxidant system under stress conditions.

### Trehalose/T6P as a signaling molecule and crosstalk with sugars and hormones

Despite being found very minutely in plants, trehalose has many roles in plant cell metabolism, contributing to abiotic stress tolerance. For instance, Sah et al. (2016) report that in most plants, trehalose serves as a signaling molecule rather than its direct involvement in abiotic stress alleviation. As such, in different living organisms, trehalose has been shown to act as a signaling molecule to regulate several metabolic events at different growth stages and shows crosstalk with other metabolites (Sah et al. 2016; Feng et al. 2019; Bao et al. 2024; Eh et al. 2025). These reports indicate that trehalose metabolism is essential for normal plant growth and development, and its biosynthesis takes place via a phosphorylated intermediate T6P; the latter level alterations are comparable with sucrose concentration, which is the main product of photosynthesis and the major transportable sugar in plants (John et al. 2017; Kosar et al. 2019). That is, the intermediate T6P in the trehalose biosynthesis pathway senses the available sucrose and thus directly affects the response to harsh environmental situations. This is justified as T6P, trehalose, and/or one or more enzymes of their biosynthetic pathway are part of complex interaction networks concerted with hormone and sugar-induced signaling pathways, which may have a role at different developmental stages (Kosar et al. 2019). In addition, T6P is proposed to be implicated in the regulation of the SnRK1(SNF1/AMPK group of protein kinases) activity, which is a central modulator of energy and sugar homeostasis: T6P at low concentrations has been indicated to inhibit SnRK1 in vivo and in vitro ( Waqas et al. 2026). For example, in *Arabidopsis*, T6P is a signaling molecule that regulates sucrose content and, hence, adjusts its level within the cell (Ponnu et al. 2011; Kosar et al. 2019). Also, the sugar-signaling metabolite T6P was increased in leaf, cob, and kernels at silking, and showed a regulatory function in source and sink tissues in response to salinity treatment (Iordachescu and Imai 2011; Henry et al. 2015). Similarly, a study by Ibrahim and Abdellatif (2016) supports trehalose’s role as a signaling molecule that induces wheat plants to enhance the synthesis of non-enzymatic antioxidants to scavenge ROS and thus minimize the damaging effects of water stress. The mechanism is that trehalose, as a signaling molecule, induces ROS production in plants under abiotic stresses, which sends a signal to promote enzymatic antioxidants to reduce ROS levels that are triggered by the stress. Additionally, drought stress inhibited the trehalose synthesis pathway in rose petals, which was alleviated by exogenously applied trehalose or T6P; the alleviating effects were by promoting carbohydrate accumulation, secondary metabolites, and lignin in petals (Bao et al. 2024). The results indicate that T6P or trehalose signaling is possibly related to these positive impacts and contributes to improved drought tolerance of rose plants, which maintains the quality of rose flowers under drought conditions. Therefore, the signaling function of trehalose and T6P, integrating development and metabolism about carbon supply, might be more essential than the other suggested protective or osmotic functions, although in some tissues and plants, the latter roles may be important and cannot be excluded (Delorge et al. 2014; Lunn et al. 2014).

From the studies carried out by Debast et al. (2011) on potato, Henry et al. (2015) on maize, Blanford et al. (2024) on rose and the reviews by John et al. (2017) and Onwe et al. (2022), the following conclusions have been reached: a) TPS1 and/or T6P are major players in signaling pathways and gene modulation during seedling development and stress response, ii) trehalose as a stress response and signal plays a vital protective role during abiotic stress, and iii) crosstalk between trehalose and stress-responsive secondary metabolites that participate in plant stress tolerance is evident. For instance, transgenic potato plants with altered T6P contents were developed to find out whether T6P has a signaling role in the tubers. The work demonstrated that transgenic potato lines with elevated T6P contents exhibited decreased contents of starch and ATP, and stimulated respiration rate, indicative of high metabolic activity (Debast et al. 2011). However, potato lines with lower T6P displayed soluble sugar accumulation, increased hexose phosphates and ATP, no change in starch content, and a strong reduction in tuber yield. The study also showed that the transgenic lines with target genes of *SnRK1*, involved in the promotion of cell proliferation and growth, were downregulated, while those involved in inhibiting cell cycle progression were upregulated. The work of Debast et al. also indicated that T6P accumulation in tubers drastically postponed sprouting, while those with reduced T6P sprouted earlier than the wild type; reduced ABA level was correlated with early sprouting of tubers. In agreement, Figueroa and Lunn (2016) provided diverse lines of evidence that the link between growth, development, and carbon status to maintain sucrose levels within an optimal range is maintained. That is, in source leaves, T6P modifies sucrose levels by influencing sucrose synthesis, while in sink organs, T6P controls sucrose consumption. Moreover, the presence of interaction between sugars and plant hormones in metabolic pathways and stress response has also been suggested. This is supported by the finding that the application of trehalose upregulates the gene expression responsible for ABA biosynthesis and downregulates metabolic genes that enhance the ABA content in plants (León 2003; Feng et al. 2019). It is, therefore, clear from these findings that trehalose serves as a signaling molecule via controlling the ABA anabolic pathway, which results in ABA accumulation and enhances the salinity tolerance of tomato plants. The results provide greater insights into understanding the salinity tolerance mechanism of plants and have important significance for promoting salinity resilience and crop yield. A recent work by Wang et al. (2022) also illustrated that trehalose and ABA have synergistic impacts on root growth and stomatal closure, as ABA has been observed to stimulate the expression of *TPP* and hence increases trehalose content. Recently, Kerbler et al. (2023) and Blanford et al. (2024) similarly report T6P as an essential signaling molecule linking carbon metabolism to plant growth and development. The latter authors also indicated that TPPs play pivotal regulatory roles in regulating endogenous levels of T6P and trehalose, and T6P signaling, as well as in integrating environmental signals with plant metabolism. In conclusion, trehalose, its precursor T6P, and enzymes of its biosynthesis pathway (TPP, TPS) have a crucial role as signaling molecules. Therefore, they greatly regulate sugar metabolism as well as ABA levels, which strongly suggests crosstalk between them, resulting in a link between stress, metabolism, and development. However, the role of these metabolites in signaling, metabolism regulation, hormone levels, and plant development during stress needs further molecular elucidation and exploration.

### Induction of ion homeostasis, accumulation of osmolytes, and secondary metabolites

The capacity of plants to retain ion homeostasis in response to high salinity is a pivotal trait in determining plant adaptation to saline soil (Han et al. 2024; Islam et al. 2025). Trehalose improves *Catharanthus roseus* salinity resistance due to trehalose’s ability to reduce the Na^+^/K^+^ ratio in salinity-stressed plants (Chang et al. 2014). In this work, trehalose treatment increased sugar accumulation, net photosynthesis rate, transpiration rate, and stomatal conductance, suggesting that the trehalose-enhancement of sugars (i.e., trehalose, sucrose, fructose, glucose, soluble sugars, free amino acids, and alkaloids) allowed the salinity-stressed plants to maintain a status of water relations suitable for growth and photosynthesis under high saline situations. It can also be concluded from this research that trehalose efficiently induces osmolyte accumulation, which plays a part in the salinity tolerance mechanism. Further evidence supporting trehalose’s role in accelerating ion balance under saline conditions and hence enhancing crop resilience is that transgenes with increased trehalose content can maintain a greater selectivity for K^+^ over Na^+^ uptake in rice roots (Fig. 4), which improved the rice salinity tolerance when grown under high salinity (Garg et al. 2002). Another study by Garcia et al. (1997) also illustrated that exogenous trehalose remarkably decreased Na^+^ accumulation triggered by salinity, suppressed *salT* (an osmotically regulated gene) expression, and enhanced growth in rice. Here, the proposed mechanism underpinning rice salinity tolerance by trehalose application is trehalose-induced Na^+^ exclusion from root cells by protecting membrane transport proteins in response to salinity stress. Furthermore, exogenous trehalose overcomes salinity-induced reduction in growth and reproduction of Arabidopsis under high salinity by ionic homeostasis maintenance, i.e., retained higher K^+^ and K^+^/Na^+^ ratios in the leaf and stem of the inflorescence (Yang et al. 2014). In this investigation, it is also illustrated that trehalose was capable of restricting Na^+^ transport from leaves to the inflorescence stem. Phan and Van Dijck (2019) similarly indicated that the mechanism by which trehalose can protect plants against stress is through its protection of proteins, membrane integrity, and ion pumps, which help to prevent Na^+^ uptake into chloroplasts. Taken together, trehalose has been found to regulate the K^+^/Na^+^ ratio and modify osmolyte metabolism and accumulation under saline conditions. However, the implicated molecular mechanisms in how trehalose regulates ionic balance under salinity imposition need further research and exploration.

In response to saline conditions, a strategy has emerged in plants to adjust the osmotic potential of the cell by overproduction of compatible solutes, such as sugars, proline, and glycinebetaine (Kosar et al. 2019; Islam et al. 2025). Compatible solutes are important osmotic regulators as they can significantly lower the cell osmotic potential, providing the required water potential gradient for water absorption and hence turgidity retention, and they also stabilize cellular proteins and other cell components against the harmful effects of high salinity (Iordachescu and Imai 2011). Further evidence indicating trehalose’s implication in accelerating osmolyte levels under saline conditions is provided in this section. For instance, exogenous trehalose increases the soluble sugar content in tomato seedlings by upregulating the expression of genes encoding enzymes responsible for their biosynthesis (Feng et al. 2019). Additionally, trehalose also alters soluble sugar accumulation and distribution by upregulating the expression of genes related to sugar transporters (Feng et al. 2019). In quinoa and rice plants, trehalose supply stimulates the production of compatible osmolytes such as proline, trehalose, total soluble sugars, and free amino acids under salinity stress (Abdallah et al. 2016, 2020), indicating trehalose’s capacity to upregulate osmolyte and secondary metabolite accumulation that are involved in osmoregulation and protective functions as well. Also, trehalose-induced compatible osmolytes and secondary metabolites production in maize (Henry et al. 2015), cucumber (Huang et al. 2023), Indian mustard (Islam and Mohammad 2021; Islam et al. 2023), rice (Joshi et al. 2020), and tomato (Yang et al. 2022a) in response to high salinity has been documented. Externally applied trehalose promoted starch accumulation, soluble sugar, and lignin in the rose petals during drought stress, alleviating drought impacts and enhancing tolerance (Bao et al. 2024). Collectively, these solutes participate in cell osmotic balance regulation, ROS scavenging as non-enzymatic antioxidants, as well as cellular structure integrity protection, and thereby contribute to crop salinity stress alleviation and tolerance. It can also be inferred from these works that trehalose upregulates genes encoding enzymes that catalyze the production of these solutes. However, the mechanism by which trehalose activates stress-responsive genes/proteins in response to saline conditions needs to be deciphered.

### Trehalose as a macromolecular protector

An important strategy plants adopt to cope with various abiotic stresses is the biosynthesis and accumulation of excessive compatible solutes, which contribute to osmotic adjustment for maintaining continued water absorption. These osmolytes are called compatible osmolytes as they, even at high concentrations in the cytoplasm, are compatible with the cellular metabolism and function to balance the external lower water potential of the soil. Except in certain resurrection plants (e.g., *Selaginella lepidophylla*) and specific organs upon stress exposure, trehalose is hardly detectable in most plants, and therefore it is not considered a solute that contributes to osmotic adjustment under stress conditions (Figueroa and Lunn 2016; Kahraman et al. 2019). It is most likely that other osmolytes take over this function, whereas trehalose serves as a cellular constituent protectant and signaling molecule under salinity imposition. Trehalose, hence, is reported to be a remarkably effective protectant due to its roles in stabilizing the cellular membranes, proteins, and protein complexes, and cell components under abiotic stresses (Figueroa and Lunn 2016; Sah et al. 2016; Onwe et al. 2022). Reportedly, these special functions of trehalose are related to its physicochemical properties because trehalose can protect protein and membrane structure deformation by replacing the hydrogen bonds of water molecules that form the water film surrounding these cellular structures as well as creating a gel phase in the cells exposed to dehydration, thereby protecting cells against drought and high salinity (Avonce et al. 2006; Figueroa and Lunn 2016). These protective properties of trehalose are reported to be superior to those of other sugars, such as sucrose, making it an ideal stress protectant (Iordachescu and Imai 2011; Yang et al. 2014; Figueroa and Lunn 2016). In support, an in vitro experiment by Magazù et al. (2012) illustrated the effectiveness of trehalose in protecting proteins from degradation and even outcompetes sucrose. In addition, lipid-based cells maintain their status of fluidity under stress conditions with a trehalose stabilizing role, preventing membrane leakage and fusion (Crowe et al. 1984; Iturriaga et al. 2009). Abdallah et al. (2016) also suggested that trehalose’s effective mitigating impact against salinity stress in two rice cultivars was likely due to its role in stabilizing antioxidant enzymes and proteins, as well as protecting other cellular structures, which suggests the protective function of trehalose macromolecules under stress. Additionally, interestingly, trehalose was one crucial alternative osmoprotectant to proline in rice cultivars exposed to NaCl stress (Garcia et al. 1997). In this investigation, while proline caused inhibitory effects, trehalose reduced Na^+^ accumulation, loss of chlorophyll, and enhanced growth under salinity. The protective role of trehalose is explained by its excellent capacity to stabilize lipid bilayer and enzyme functioning, thus preserving their integrity and role under salinity stress (Garcia et al. 1997). Thus, the study showed the importance of trehalose for rice more than proline under saline stress. Consistently, proline proposed functions in plants under salinity stress are argued by Mansour and Ali (2017) as proline accumulation showed a negative correlation with salinity tolerance, and proline functions are not always confirmed under saline imposition in many plants.

Trehalose exogenous application also provides beneficial protection of the cellular macromolecules in plants facing salinity stress. For instance, trehalose via its direct interaction with *cis* double bonds of the cellular macromolecules has been indicated to protect unsaturated fatty acids against oxidative stress, preventing protein aggregation, and limiting the acetylation of lysine amino groups (Kuczynska-Wisnik et al. 2024). Another mechanism by which trehalose shows its capability to protect the cellular macromolecules and membranes is achieved through trehalose’s direct suppression of ROS damage effects, as trehalose itself is proposed as an antioxidant effector or indirectly activates the antioxidant defense systems that scavenge ROS (Luo et al. 2008; Onwe et al. 2022). The work of Luo et al. (2008) demonstrated that trehalose plays a direct role in scavenging ROS (O_2_^−^, H_2_O_2_) in wheat under heat stress, which is possibly the case under saline conditions, although it requires further confirmation. Therefore, minimizing cellular ROS levels induced by trehalose protects membranes, proteins, and nucleic acids from oxidative damage triggered by saline imposition. Further, Fordil and Khan (2022) reported trehalose alleviation of the harmful impacts of NaCl stress on ten Pakistani wheat varieties, showing trehalose capacity to act as an osmoprotective agent, which reduces the damage caused by salinity. Trehalose’s protective function is also reported by Figueroa and Lunn (2016) since trehalose accumulation in chloroplasts preserves their functionality under salinity stress, suggestive of trehalose stabilizing impact on the chloroplast architecture. Similarly, trehalose treatment increased the net photosynthesis rate and enhanced *Catharanthus roseus* salinity tolerance, which was partly attributed to trehalose’s protective effects on the photosynthetic apparatus (Chang et al. 2014). Moreover, trehalose priming has been proposed to protect the macromolecular structures of maize plants against the destabilizing effect of salinity stress, improving maize performance under high salinity (Zeid 2009). Based on the above evidence, it is therefore obvious that trehalose effectively protects cellular macromolecules against the hazardous effects of salinity imposition, thereby improving salinity resilience.

## Genetic engineering of trehalose-encoding genes (*TPS* and *TPP*)

To develop salinity tolerance in crop plants, engineering a trehalose biosynthetic pathway is one of the most promising approaches because trehalose plays a chief role in regulating various tolerance mechanisms (Jiang et al. 2014; Sah et al. 2016; Feng et al. 2019; Joshi et al. 2020; Ye et al. 2023). The development of transgenic lines with trehalose biosynthesis genes provides numerous possibilities for enhancing crop salinity resilience and thereby improving yield production. These transgenes also showed favorably altered nutritional metabolites of seeds and tubers. However, some transgenic plants overexpressing trehalose biosynthesis genes and overaccumulating trehalose have been reported to be adversely affected (Zhang et al. 2005; Delorge et al. 2014; John et al. 2017). Therefore, trehalose accumulation by biosynthesis gene overexpression should be precisely controlled to optimal subcellular levels to avoid such adverse effects on plants. In addition, trehalose naturally accumulates in a broad variation among plants and genotypes, indicating that trehalose’s optimal level of accumulation is species/genotype dependent. This also needs to be considered in transgenic approaches seeking to modify biosynthesis genes (*TPS*, *TPP*) to improve salinity tolerance. Several options are proposed to avoid such phenotypic alterations in response to engineering trehalose biosynthesis genes: (a) a tight regulation between biosynthesis and degradation of T6P and/or trehalose at the cellular level should be balanced as an imbalance in the level of T6P and/or trehalose has been reported to result in these aberrant phenotypes (Bánfalvi 2019; Waqas et al. 2026), (b) overexpression of trehalase is suggested to regulate trehalose metabolism and avoid trehalose overaccumulation (Delorge et al. 2014), and (c) regulation of the introduced genes by using stress-inducible promoters and limitation of *TPS* and *TPP* expression to certain subcellular levels (Figueroa and Lunn 2016). These options should be considered in gene manipulation techniques to enhance trehalose production in crop plants without undesirable effects. The main results of various investigations that indicate the importance of trehalose biosynthesis gene transformation in modulating transgenic crop plant responses to saline conditions are reviewed and summarized in Table 1.

**Table 1:** Transgenic crop species expressing trehalose biosynthesis genes that enhance tolerance to saline conditions

Gene	Origin	Transgenic crop	Remarks	References
*TPS*	*Oryza sativa*	*Oryza sativa*	*TPS* gene was constitutively expressed in the leaves of two rice cultivars, contrasting in salinity tolerance, under stressed and non-stressed conditions, treated and untreated with validamycin A (a competitive inhibitor of trehalase enzyme). Higher transcript amounts were obtained in the presence of validamycin A. Salinity tolerant cultivar showed increased *TPS* transcript amounts relative to the sensitive one. Upregulation of *TPS* led to trehalose accumulation and increased gene expression that is responsible for producing osmoprotectants, because trehalose was too low to serve as an osmoprotectant. The study also revealed that the *TPS* expression pattern was consistent with the increase in TPS activity, confirming that the expression of the *TPS* gene was upregulated in response to salinity and validamycin treatments, and evidenced its important role in crop salinity tolerance.	Abdelgawad et al. (2014)
*TPS*,* TPP*	*Solanum Lycopersicon*	*Solanum Lycopersicon*	Salinity upregulated the expression of *TPS* in tomato, and exogenous trehalose increased the endogenous trehalose, which caused a negative feedback regulation and suppressed the *TPP* expression. The study indicated that *TPS*, *TPP*, and trehalose exhibited differential expressions under salinity stress. The result suggests that the trehalose metabolic pathway could directly affect the salinity tolerance of crop plants.	Feng et al. (2019)
*otsA*, *otsB*	*Escherichia coli*	*Oryza sativa*	Overexpression of these genes resulted in transgenic lines exhibiting sustained plant growth, increased trehalose, high soluble sugars, less oxidative damage, elevated capacity for photosynthesis, and ion homeostasis relative to wild type under salinity stress. The study argued against the primary role of trehalose as a compatible solute, as a low level of trehalose was obtained in transgenic rice plants. The work revealed that engineering crops for the overproduction of trehalose resulted in improved salinity tolerance and productivity.	Garg et al. (2002)
*AmTPS9A*	*Avicennia marina*	*Arabidopsis thaliana*	*AmTPS9A*-overexpressing *Arabidopsis* showed increased salinity tolerance by upregulating the expression of genes encoding ion transporters that mediate Na^+^ efflux from the roots of transgenic Arabidopsis under NaCl treatments.	Song et al. (2024)
*TPS* (*otsA*)	*Escherichia coli*	*Nicotiana tabacum*	Transgenic plants produced small amounts of trehalose, maintained leaf turgidity and fresh weight, had more efficient seed germination, and showed a drastic delay in leaf withering and chlorosis under 250 mM NaCl. It appears that trehalose did not participate in osmotic adjustment but rather may have had a protective or signaling role.	Jun et al. (2005)
*TPS*	*Tamarix hispida*	*Tamarix hispida*	*ThTPS* overexpression was induced by salinity treatment, which promoted the biosynthesis of trehalose, decreased the accumulation of O^2−^ and H_2_O_2_, and thus improved the *T. hispida* salinity resistance, suggestive of enhancing the antioxidant defense system by trehalose under salinity.	Wang et al. (2020b)
*TPSP*	*Escherichia coli*	*Oryza sativa*	*TPSP* transgenic plants retained higher yield, RWC, chlorophyll content, trehalose content, K^+^/Na^+^ ratio, stomatal conductance, efficient photosynthesis, and rice seed nutritional levels compared with the wild type under salinity imposition. This finding confirms that trehalose acts as a signaling molecule that alters other metabolic switches, resulting in significant changes in the levels of other tolerance traits in transgenic plants, which support salinity tolerance under stress conditions.	Joshi et al. (2020)
*TPS* (*TSase*)	*Grifola frondosa*	*Nicotiana tabaccum*	Transgene tobacco showed increased trehalose, high water content, chlorophyll content, and antioxidant enzyme activities, enhanced salinity tolerance with obvious morphological changes, but no growth inhibition.	Zhang et al. (2022b)
*AtTPPD*	*Arabidopsis thaliana*	*Arabidopsis thaliana*	Plants overexpressing *AtTPPD* (its product enzyme AtTPPD is chloroplast-localized) produced high levels of starch and soluble sugars, which contributed to salinity tolerance. The result suggests that trehalose metabolism possibly regulates various biological processes by relaying the redox status of different cellular compartments. Also, under salinity stress, the transcript levels of the chloroplastic *AtTPPD* were highly increased, while *tppd* mutants were hypersensitive, indicative of the trehalose protective role of the thylakoid membranes when accumulated in the chloroplasts under salinity stress	Krasensky et al. (2014)
*IbTPS*	*Ipomoea batatas*	*Nicotiana tabaccum*	Transgenic tobacco overexpressing the *IbTPS* gene resulted in significantly higher salinity tolerance, trehalose, and proline content than wild type under high salinity. Several stress tolerance-responsive genes were upregulated, suggesting that the *IbTPS* gene may enhance salinity tolerance by increasing the level of trehalose and proline and modulating the expression of stress tolerance-related genes.	Jiang et al. (2014)
*TPS1*	*Saccharomyces cerevisiae*	*Lycopersicon esculentum*	*TPS1* transgenic tomato plants displayed morphological changes such as thick shoots, rigid dark-green leaves, erect branches, and aberrant root development. The transgenic *TPS1* tomato had leaves with high chlorophyll and starch contents. The study revealed that engineering tomato through trehalose biosynthesis showed increased salinity tolerance and yield in the presence and absence of stress.	Cortina and Culianez-Macia (2005)
*TPS3*	*Oryza sativa*	*Oryza sativa*	ABA and salinity imposition upregulate *OsTPP3* overexpression, which elevates trehalose content and improves salinity tolerance in rice seedlings. The work also revealed that the knockout of *OsTPP3* reduced rice salinity tolerance associated with a decline in trehalose level. Trehalose application enhanced the salinity tolerance of the *tpp3* mutant plant, indicating trehalose’s crucial role in improving crop salinity tolerance.	Ye et al. (2023)
*OsTPS1*	*Oryza sativa*	*Oryza sativa*	Overexpression of *OsTPS1* improved the resilience of rice seedlings to salinity stress by elevating the content of trehalose and proline and upregulating stress-related genes under saline conditions relative to the wild type.	Li et al. (2011)
*Ubi1: TPSP*	*Oryza sativa*	*Oryza sativa*	Overexpression of *Ubi1: TPSP* induced a significant increase in endogenous trehalose and soluble sugars in transgenic rice and improved its salinity resistance to 150 mM NaCl.	Redillas et al. (2012)
*OsTPP1*	*Oryza sativa*	*Oryza sativa*	*OsTPP1* expression was upregulated under salinity, and its overexpression enhanced seed germination and trehalose levels, triggered stress-responsive genes, and upregulated the expression of *OsTPS1*, which contributed to rice salinity tolerance. The study indicates the potential use of *OsTPP1* in the salinity stress engineering of crops of other crops.	Ge et al. (2008)
*otsA* (*TPS*)	*E. ctdi*	*Brassica campestris*	Overexpression of *OtsA* in Chinese cabbage resulted in transgenic plants showing retained turgidity and photosynthesis rate, delayed necrosis, and remarkably improved salinity tolerance, but exhibited altered phenotypes, including stunted growth and aberrant root development relative to wildtype when subjected to 250 mM NaCl stress.	Park et al. (2003)
*AtTPS1*	*Arabidopsis thaliana*	*Nicotiana tabacum*	Transgenic seeds germinated in different NaCl concentrations, scoring salinity tolerance compared with untransformed plants, confirming that *AtTPS1* overexpressing lines stimulated crop tolerance to NaCl stress.	Almeida et al. (2005)
*TPS1*, *TPS2*	*Saccharomyces cerevisiae*	*Arabidopsis thaliana*	Lines overexpressing the *TPS1*-*TPS2* construct accumulated trehalose and exhibited a significant increase in salinity resilience with no morphological or growth alterations, while plants overexpressing the *TPS1* alone exhibited modified growth, color, and shape. *TPS1-TPS2* overexpressing lines were insensitive to glucose, confirming the proposed role of trehalose/T6P in modulating sugar sensing and carbohydrate metabolism.	Miranda et al. (2007)
*ZmTPS*	*Zostera marina*	*Oryza sativa*	Transgenic rice plants overexpressing *ZmTPS* showed increased endogenous trehalose and tolerance to 150 mM NaCl relative to untransformed control plants. Transgenic plants had no phenotypic aberrations. The work also illustrated that the transformed *ZmTPS* gene can be transmitted stably from the parent to the progeny in transgenic rice.	Zhao et al. (2013)
*OsTPS8*	*Oryza sativa*	*Oryza sativa*	Constitutive overexpression of *OsTPS8* enhanced soluble sugars and regulated the expression of genes involved in ABA signaling via *SAPK9* regulation, which promoted suberin deposition in the root and reduced Na^+^ content in the shoot and root; this, in turn, improved salinity tolerance and grain yield in rice with no aberrant changes in plant growth and development under saline stress compared with the wild type.	Vishal et al. (2019)
*OsTRE1*	*Oryza sativa*	*Oryza sativa*	Overexpressing the trehalase gene, *OsTRE1*, exhibited considerable increases in trehalase activity and remarkable declines in trehalose levels under 150 mM NaCl, with little change in the levels of other soluble sugars. Transgenic plants exhibited enhanced salinity resilience with no morphological alterations or growth defects, suggesting the involvement of *OsTRE1* in salinity tolerance in rice. Trehalose accumulation appears not to be a prerequisite for better adaptation to salinity stress, but rather possibly the endogenous trehalose biosynthesis pathway.	Islam et al. (2019)
*otsA* (*Ubi1: TPSP*)	*Escherichia coli*	*Oryza sativa*	Transgenic rice produced by the transformation of a gene encoding a bifunctional fusion (*TPSP*) of *TPS* and *TPP* of *Escherichia coli* elevated trehalose levels in the leaves and seeds, reduced the accumulation of potentially deleterious T6P, displayed no growth inhibition or visible morphological alterations, and enhanced tolerance to salinity stress.	Jang et al. (2003)
*TPS*,* TPP*	*Saccharomyces cerevisiae*	*Medicago sativa*	When both genes (*TPS*,* TPP*) were fused and expressed, transgenic alfalfa plants displayed improved growth, biomass production, and a significant increase in salinity tolerance. TPS-TPP fusion protein is promising for crop salinity tolerance and enhanced yield under saline conditions.	Suarez et al. (2009)
*otsA* (*TPS*)	*Mesorhizobium* sp. *CCBAU25338*	*Arachis hypogaea*	Overexpression of trehalose synthesis genes *otsA* in the peanut-nodulating rhizobium *Mesorhizobium sp. CCBAU25338* enhanced the salinity stress tolerance and nitrogen-fixing capacity of the rhizobium strain, as well as increased endogenous trehalose, agronomic traits, and lowered oxidative damage in peanuts under salinity conditions.	Liu et al. (2025)
*GmTPP*	*Glycine max*	*Glycine max*	The expression levels of *GmTPPs* were upregulated in response to saline-alkali stress in soybean and were tissue-specific (i.e., flowers, stems, roots, nodules, shoots, leaves, pods, and seeds), suggesting that they trigger developmental processes and stress responses that confer stress resilience.	Shao et al. (2023)
*ArTPS*	*Anoectochilus roxburghii*	*Escherichia coli*	*ArTPS* overexpression in *E. coli* showed higher suitability under 300 mM NaCl stress, playing a key role in polysaccharide and glycoside metabolism. This indicates that the *ArTPS is* involved in increasing the T6P and trehalose contents, resulting in higher tolerance to salinity stress.	Yang et al. (2023)

## Trehalose exogenous application

The approach of trehalose exogenous application is based on the fact that when plants are overwhelmed by salinity stress and the perturbation exceeds the plant’s ability to deal with it or emerges from different sources in addition to the fact that trehalose is detected in very low level in plants, an exogenous supply of trehalose or T6P could be an efficient strategy to improve several crop plant performance under saline soil (Mostofa et al. 2015; Feng et al. 2019; Abdallah et al. 2020; Fordil and Khan 2022; Bao et al. 2024). Under stressful saline conditions, exogenous trehalose could improve crop salinity tolerance, which most likely functions as a modulator of the biological processes implicated in crop salinity stress responses and adaptation. Trehalose external applications have been reported to have the advantages of being available, absorbable, transportable, cheap, and non-toxic (Lin et al. 2017). A tabulated review of the responses of crop plants contrasting in salinity resistance to exogenously applied trehalose or T6P under saline conditions is given in Table 2. However, these trehalose-induced mitigative effects are not unique and similar but depend on various factors. Therefore, conclusions are drawn based on the results presented in Table 2 for consideration when trehalose is supplied externally. These include (a) the trehalose impacts seem to rely on the concentration used, and they are species-specific. It is, therefore, recommended to justify trehalose concentration and other factors regulating its efficacy for each plant species/genotype, (b) roots efficiently absorb trehalose which is easily transported to the shoot and leaves to trigger several adaptive mechanisms functioning as major defensive responses to saline stress, (c) internal trehalose content depends on plant species/genotype, different providing approaches, tissue and cell type, and plant developmental stage at which trehalose applied. Therefore, these conditions should be considered when interpreting the results and drawing conclusions, (d) T6P and trehalose foliar spray showed functional differences in rose plants under drought stress, as the response of gene expression to T6P or trehalose was different under stress. This also needs to be explored under saline imposition, (e) trehalose toxicity to plants, when applied in high concentrations, depends on a link between T6P, SnRK1, bZIP11, sucrose, and starch, and (f) The salinity’s drastic impacts on the expression of the trehalose pathway genes and T6P showed remarkable differences between source and sink tissues through T6P impact on SnRK1 activity. This impact occurs through differential effects on SnRK1 marker genes between source and sink tissues, possibly suggesting unrelated functions.

**Table 2:** Exogenous application of trehalose improves crop performance by triggering different adaptive mechanisms in response to saline conditions

Crop species	Mode of application	Trehalose concentration (mM)	Response	References
*Oryza sativa*	Seed priming	0, 25	Alleviated the harmful effects of salinity stress, increased soluble sugars and proline contents contributing to osmotic adjustment, and elevated internal trehalose, stimulating the antioxidant enzyme activities in two rice varieties under salinity imposition.	Abdallah et al. (2016)
*Chenopodium quinoa Masr 1*	Foliar spray	0, 2.5, 5	Growth, total soluble sugars, proline, free amino acids, photosynthetic pigments, yield attributes, antioxidant enzyme activities, and seed nutritional value were improved in quinoa plants by trehalose foliar spray under salinity stress, more so when compost was added to the soil.	Abdallah et al. (2020)
*Triticum aestivum*	Foliar spray	0, 5, 10, 15 (mg L^− 1^)	Trehalose foliar spray stimulated growth parameters, trehalose content, proline content, and antioxidant enzyme activities in response to salinity imposition; these effects were more pronounced with 15 mg L^− 1^.	AL-Zewany and Al-Semmak (2017)
*Avicennia marina*	Added to the Hoagland solution	0, 20	Exogenous trehalose enhanced salt resilience by increasing Na^+^ efflux from the leaf salt gland and root, which reduces the Na^+^ content in the root and leaf.	Song et al. (2024)
*Arabidopsis thaliana*	Added to liquid cultures	0, 30	Levels of trehalose, sucrose, and starch were elevated in response to trehalose treatment. Two-dimensional gel electrophoresis identified nine altered proteins, four of which were responsible for detoxification or stress responses. This work indicates that the external supply of trehalose acted as a regulator of genes involved in responses to abiotic stresses. As exogenous trehalose altered transcript levels of several processes related to tolerance mechanisms, we suggest that trehalose, or metabolites derived from its biosynthesis pathway, are key modulators of gene expression in higher plants under stress.	Bae et al. (2005a,b)
*Catharanthus roseus*	Added to the nutrient solution	0, 10, 30, 50	Supplementation of trehalose to the nutrient solution markedly alleviated the inhibitory effects of salinity on plant growth, relative water content, and photosynthetic rate by decreasing Na^+^, increasing K^+^, soluble sugars, free amino acids, and leaf gas exchange in leaves under 250 mM NaCl. The regulatory role of exogenous trehalose in stimulating salinity tolerance was optimal at 10 mM, while higher concentrations adversely affected plant growth. It appears that exogenous trehalose acted as a signal to induce salinity-stressed plants to efficiently raise internal compatible solutes to regulate water loss and turgidity, leaf gas exchange, and ion homeostasis under salinity.	Chang et al. (2014)
*Solanum Lycopersicon*	Added to the nutrient solution	0, 1, 2, 3, 5, 10	Exogenous trehalose increased growth characteristics, chlorophyll content, proline, internal trehalose, and antioxidant enzyme activities, while decreasing lipid peroxidation, which effectively enhanced tomato salinity tolerance in response to 200 mM NaCl stress. The study also showed that the starch content declined and soluble sugars elevated as trehalose modulated the gene expression of starch and soluble sugar metabolism, induced the upregulation of sugar transporter genes, and those related to the synthesis and metabolism of ABA. It is obvious that trehalose’s role in regulating the above processes significantly modulates sugar accumulation, content, and distribution, thereby improving plant stress resilience. Notably, 2 mM provided optimal responses, and higher concentrations showed decreased responses.	Feng et al. (2019)
*Triticum aestivum*	Added to a growth medium	0, 10, 50	Trehalose supply significantly stimulated several growth parameters of ten wheat varieties under 150 mM NaCl stress.	Fordil and Khan (2022)
*Brassica juncea*	Foliar spray	0, 10	Leaf-applied trehalose increased salinity tolerance and yield by enhancing ion homeostasis, photosynthesis efficiency, antioxidant defense mechanisms, chlorophyll content, osmolyte accumulation, stomatal aperture, cell viability, and ROS scavenging under salinity stress.	Islam et al. (2025)
*Solanum Lycopersicon*	Foliar spray	0, 5, 10, 25	Trehalose application scavenges ROS by enhancing the activities of antioxidant enzymes and their related gene expression, improves growth, biomass production, proline, and glycine betaine content, increases K^+^ and K^+^/Na^+^ ratio, upregulates the expression of trehalose genes (*SlTPS1*, *SlTPS5*, *SlTPS7*, *SlTPPJ*, *SlTPPH*, and *SlTRE*), and the activity of enzymes involved in its metabolic pathway, which in turn altogether stimulated tomato salinity tolerance. Trehalose at 10 mM was the best mitigation concentration, while 25 mM caused leaf damage and adversely affected plant growth under salinity.	Yang et al. (2022a)
*Solanum Lycopersicon*	Foliar spray	0, 10	Trehalose foliar supply improves photosynthetic efficiency, increases the activity of Calvin cycle enzymes, upregulates the expression of their related genes, protects the photosynthetic electron transport system, affects the expressions of *SlSOS1*, *SlNHX*, *SlHKT1.1*, *SlVHA*, and *SlHA-A*, which retain ion homeostasis, induces stomatal opening, and alleviates salt-induced damage to the chloroplast membrane and structure under salinity. The study shows that trehalose renders salinity tolerance by upregulating the processes involved in mitigating salinity toxicity.	Yang et al. (2022b)
*Zea mays*	Added to the nutrient solution	0, 10	Trehalose application improved the performance of two maize genotypes in response to 150 mM NaCl by decreasing the Na^+^/K^+^ ratio, ROS, lipid peroxidation, methylglyoxal, and increasing leaf trehalose, salinity tolerance, indicative of regulating antioxidant and glyoxalase systems as well as ion homeostasis.	Rohman et al. (2019)
*Triticum aestivum*	Foliar spray	0, 10, 50	Trehalose enhanced wheat growth and accumulation of compatible solutes (i.e., glucose, sucrose, trehalose, phenolic compounds, total soluble sugars) but decreased lipid peroxidation, hydrogen peroxide, and lipoxygenase activity of salinity-stressed wheat plants. It seems that antioxidant compounds and compatible osmolytes play a crucial role in enhancing wheat performance under salinity by counteracting oxidative damage and, hence, protecting cellular structures.	Sadak (2019)
*Cucumis sativus*	Added to the nutrient solution	0, 0.05, 0.2, 0.4, 0.6, 0.8, 1%	Hydrogen-induced cucumber seed germination was done by enhancing enzyme activity and gene expression levels of trehalose metabolism-related genes, which in turn increased the endogenous trehalose content. Although the molecular mechanism and hydrogen crosstalk with other signaling molecules in inducing seed germination are not clear, this work provides new insights concerning the roles and interactions of hydrogen and trehalose during seed germination and confirms one of the fundamental trehalose roles. That is, trehalose as a key regulator of carbon metabolism, largely influences plant growth and development.	Huang et al. (2023)
*Brassica juncea*	Foliar spray	0, 10, 20, 30	Under the non-saline conditions, trehalose application improved Indian mustard growth characteristics and yield by enhancing osmolyte accumulation and enzyme activities, thereby reducing the ROS content, while promoting the content of photosynthetic pigments, gas exchange, mineral acquisition, and root cell viability. These improvements were more pronounced by 10 mM, while other trehalose concentrations were either equally or less effective.	Islam and Mohammad (2021)
*Brassica juncea*	Foliar spray	0, 10	Foliar spraying of trehalose stimulates the enzymatic antioxidant activities, compatible solute content, water status, and membrane permeability while decreasing ROS production, lipid peroxidation, and Na^+^ levels under salinity stress. These effects resulted in improved Indian mustard growth, ion homeostasis, photosynthesis, yield, and salinity resilience.	Islam et al. (2023)
*Oryza sativa*	Added to the nutrient solution	0, 10	Trehalose protects rice seedlings against NaCl stress by increasing the level of internal trehalose, which effectively reduces ROS accumulation, elevates nonenzymatic antioxidants, and co-activates the antioxidative and glyoxalase systems.	Mostofa et al. (2015)
*Arabidopsis thaliana*	Added to the nutrient solution	0, 0.5, 1, 5	Trehalose improved *Arabidopsis* salinity tolerance by retaining K^+^ content and a high K^+^/Na^+^ ratio, decreasing Na^+^ level, enhancing internal soluble sugars, and the activities of antioxidant enzymes in plant tissues under NaCl imposition. Thus, it is evident that trehalose regulates plant redox state and ionic distribution under high salinity. Trehalose at 1 mM showed the best salinity tolerance responses.	Yang et al. (2014)
*Triticum aestivum*	Plant spray	0, 10	Trehalose promoted the growth parameters, yield, leaf anatomical features, endogenous trehalose, amino acid, sugar, total carbohydrate, and total soluble protein contents in 4 wheat cultivars contrasting in their salinity tolerance under 200 mM NaCl.	Mohamed et al. (2018)
*Oryza sativa*	Added to the nutrient solution	0, 10	Trehalose supplement to 200 mM NaCl-stressed rice cultivars, contrasting in salinity resilience, did not mitigate the growth reduction during stress, while during recovery plants previously supplied with trehalose displayed a significantly higher growth recovery compared with plants that received only NaCl treatment. This mitigative effect was due to reduced Na^+^/K^+^ ratio and H_2_O_2_ level, and enhanced ascorbate peroxidase activity. The impact was more pronounced in the salinity-sensitive cultivar.	Nounjan and Theerakulpisut (2012)
*Oryza sativa*	Foliar spray at the tillering stage	0, 50, 100, 150	Trehalose enhanced RWC, chlorophyll, soluble sugars, reproductive tillers per plant, grains per panicle, 100-grain weight, percentage of filled grains per panicle, and grain yield per plant in the salt-tolerant variety relative to other varieties.	Soares et al. (2023)
*Oryza sativa*	Added to a growth culture	0, 1, 5, 10	Application of low trehalose concentrations (up to 5 mM) significantly decreased Na^+^ accumulation, overcame the growth inhibition, and enhanced the expression of the *salT* gene, while higher concentrations (10 mM) preserved the root integrity, prevented the chlorophyll damage in leaf blades, and suppressed the expression of the NaCl-induced *salT* gene. Interestingly, trehalose did not prevent salt accumulation in plant cells, but did reduce Na^+^ accumulation in laminae. It is clear that trehalose protects cellular structure under salinity, but the observed differences in response and between low and high trehalose concentrations might reflect different modes of action at various concentrations, as well as differences in the accumulation or catabolism of trehalose in different parts of the plant.	Garcia et al. (1979)
*Rosa rugosa*	Foliar spray	50 µM T6P, 20 mM trehalose	Exogenous trehalose or T6P improved the drought tolerance of rose plants by alleviating the injurious impact of drought stress, maintaining the rose flower quality, T6P and trehalose accumulation, adjusting the carbohydrate distribution, and altering the synthesis of secondary metabolites (geraniol, total flavonoids, and total anthocyanins). Foliar application of trehalose or T6P also promoted the contents of starch, soluble sugar, and lignin in the petals, pointing to the role of T6P or trehalose as a positive regulatory signal participating in enhancing the rose plant’s resilience to drought stress.	Bao et al. (2024)
*Oryza sativa*	Added to a growth culture	0, 5, 10	Exogenously supplied trehalose stimulated the growth and alleviated the harsh effects of salinity stress by reducing H_2_O_2_ and lipid peroxidation in the sensitive cultivar. However, trehalose did not have a beneficial effect on the growth of the salinity-tolerant cultivar, indicative of trehalose’s protective roles in the salt-sensitive cultivar but not in the salt-tolerant one. Although the study did not determine the endogenous trehalose content, we assume that a salinity-tolerant cultivar might have sufficient trehalose as well as other tolerance traits, and thus exogenous trehalose showed no beneficial impact on this cultivar.	Theerakulpisut and Gunnula (2012)
*Oryza sativa*	Foliar spray	0, 0.5%	Trehalose improved the salinity resilience of rice seedlings by increasing the antioxidant enzyme activities under 100 mM NaCl treatment. The study also demonstrated that salinity-induced ABA escalated the expression of *OsTPP3*, resulting in elevated endogenous trehalose, which stimulated the rice salinity tolerance to NaCl stress.	Ye et al. (2023)
*Zea mays*	Seed priming	0, 10	Trehalose alleviated the adverse impacts of high salinity on metabolic activity (Hill-reaction activity, photosynthetic pigments, and nucleic acids content), increased sugars, soluble proteins, and proline contents, leaf K^+^/Na^+^ ratio, the growth and salinity tolerance, while decreasing electrolyte leakage and lipid peroxidation of the root cells, and salinity expression detected by leaf protein banding patterns of salinity-stressed maize seedlings. It seems that trehalose upregulated various salinity stress-responsive genes that contribute to the tolerance mechanisms of maize seedlings under salinity stress.	Zeid (2009)
*Triticum aestivum*	Seed presoaking	0, 10	Seed presoaking with trehalose improved the salinity tolerance of wheat seedlings by reducing lipid peroxidation, elevating osmotic adjustment osmolytes such as amino acids (especially proline), reducing sugars, and total soluble sugars, and improving enzymatic and non-enzymatic antioxidants in wheat seedlings. This leads to maintaining the balance between pro-oxidants and antioxidants like phenols, flavonoids, proline, and soluble sugars. It is noteworthy that trehalose was more effective than mannitol.	Alhudhaibi et al. (2024)
*Glycine max*	Added to a growth culture	10 µmol/L	Trehalose treatment upregulated the expression of *TPP* genes, increasing carbohydrate and trehalose levels, while decreasing ROS content, and thus mitigating the adverse impacts caused by saline-alkaline stress, especially in the saline-alkali-tolerant genotype.	Shao et al. (2023)
*Zea mays*	Added to the hydroponic solution	0, 150	Foliar application of trehalose elevated maize biomass, antioxidant enzyme activity, reduced H_2_O_2_, and Na^+^/K^+^ ratios. Trehalose also increased total organic acids and improved the soil microenvironment for maize growth under salinity stress. Exogenous trehalose application also increased the expression levels of genes related to photosynthesis, abscisic acid signaling, and sugar metabolism, thereby mitigating the growth inhibition caused by salinity.	He and Tang (2024)
*Apium graveolens*	Foliar spray	0, 1, 5, 10,15	Trehalose foliar application mitigated the detrimental effects of salinity stress on celery by reducing excess excitation energy of the photosystem II, thereby increasing the activity of the PSII reaction center, improving photosynthetic capacity, and enhancing growth under salinity stress. A concentration of 10 mM trehalose was found to be the most effective.	Gao et al. (2026)

## Conclusions and perspectives

**Fig. 5:**
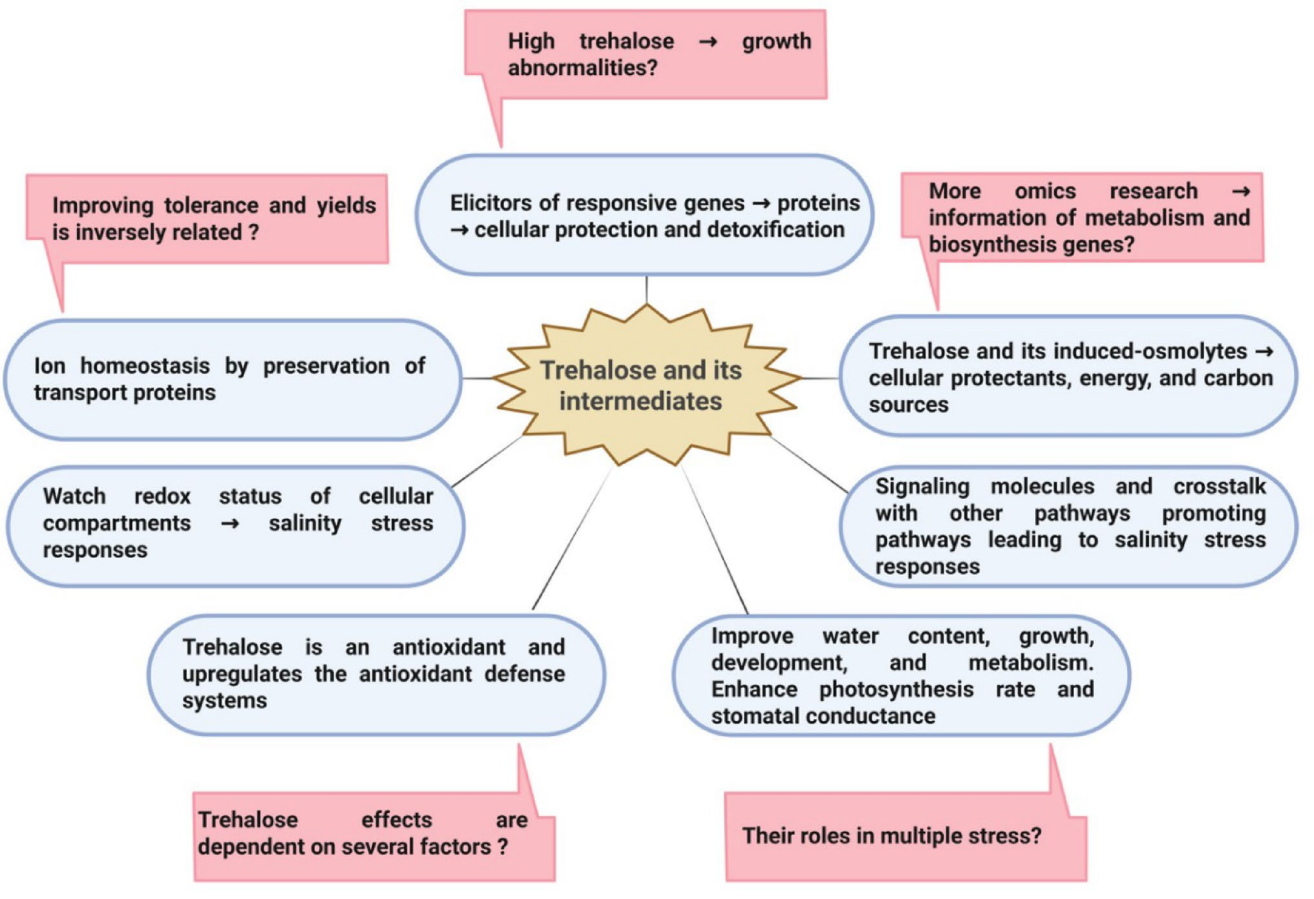
Roles of trehalose and its intermediates in improving crop plant salinity resilience and yield. Future perspectives are proposed

The current review presents various lines of evidence to raise awareness of the roles of trehalose, its precursor T6P, and biosynthetic enzymes. Figure 5 summarizes possible mechanisms by which trehalose and its intermediates promote crop plants’ salinity tolerance. Additionally, the engineering of trehalose biosynthetic genes (*TPS*, *TPP*) and the exogenous application of trehalose or T6P are discussed to assist researchers in improving crop performance and yield potential under saline conditions. This includes (a) As a signaling molecule, trehalose or T6P promotes various events and signaling pathways in response to salinity stress, (b) Trehalose or T6P functions as a modulator of genes implicated in producing stress-responsive proteins that detoxify and protect crop species from the devastating impacts of salinity stress, (c) Trehalose metabolism might watch the redox status of the cellular compartments to modulate diverse physiological processes that render salinity stress responses, (d) Trehalose and most probably trehalose-induced accumulation of osmolytes act as an energy and carbon source as well as cellular structures protectant, (e) Trehalose itself is an antioxidant, although remains incompletely understood, and upregulates other antioxidant defense system, and (f) Trehalose induces ion homeostasis, accumulation of osmolytes and secondary metabolites. In addition, exogenously applied trehalose, to compensate for the insufficient internal trehalose level, is a promising strategy to elevate internal trehalose levels and improve tolerance to saline soil in crop plants. Further, the genetic engineering of trehalose biosynthesis genes in crop plants can be a potential option to enhance their tolerance to saline conditions.

Future perspectives and research should focus on and include (a) Evidence indicates that constitutive overexpression of the trehalose biosynthetic genes (*TPP*, *TPS*) leads to growth abnormalities. We should adopt the controlled upregulation of trehalose biosynthesis to develop transgenes with improved stress tolerance, without phenotypic alterations. That is, using gene editing technology (i.e., CRISPR/Cas9) to fine-tune the promoters of *TPS* and *TPP* to ensure a balanced flux that prevents trehalose accumulation while minimizing the growth retardation often caused by excessive T6P accumulation. Additionally, regulated trehalase1 expression (*TRE1*) to modulate its activity helps maintain optimal trehalose levels and avoid toxicity, (b) Although studies have efficiently manipulated trehalose content to improve crop tolerance and productivity, trehalose roles in tolerance to multiple stresses concerning plant growth, structure, and metabolism still need huge research and exploration. That is, T6P acts as a signal for the availability of sucrose and, therefore, future strategies aim to fine-tune T6P levels by manipulating the ratio of T6P to sucrose to maintain high metabolic activity and growth under salinity stress, (c) Further intensive -omics research would be an interesting approach for providing useful information concerning trehalose metabolism during developmental stages and under saline conditions, as well as for future trehalose genetic engineering of biosynthesis genes to promote salinity tolerance and yield in crop plants, (d) Under natural saline habitats, trehalose accumulation, function, engineering, and pretreatment in crops are rare, and therefore, future research should focus on such evaluations to support our claims of the laboratory controlled results, (e) Trehalose response to saline situations greatly depends upon species/genotype, salinity intensity and type, plant organ, trehalose exogenous concentration, and developmental stage at which trehalose is applied. Therefore, these prerequisites should be taken into consideration and established to reach a plausible conclusion before recommending trehalose for agricultural practices, and (f) In response to escalating world population and climate change pressures, our ultimate goal is to enhance crop productivity required for global food security, either under favorable or poor conditions alike. However, simultaneously stimulating both crop yield and tolerance is challenging, as the mechanisms determining stress tolerance and yield are typically inversely related. In this respect, we believe that the protocol by Paul et al. (2018) to appropriately target trehalose/T6P in certain cells, tissues, and developmental stages should be given serious consideration and adopted to enhance both productivity and stress tolerance. For example, targeting the phloem to ensure carbon delivery to seeds/grains during the reproductive stage, preventing yield loss, and targeting trehalose accumulation in roots can improve water uptake and reduce Na^+^ toxicity without affecting shoot growth. This manipulation of trehalose metabolism to meet specific targets and tissues aims to stimulate yield potential and stress tolerance simultaneously, and (g) Future research should focus on quantitative synthesis (i.e., effect sizes, dose-response ranges, field vs. greenhouse). In particular, studies concerning field-level validation under saline conditions are required to translate laboratory findings into agricultural applications and thus support our claims. For example, trehalose foliar spray or seed priming has proven effective in improving chlorophyll content, antioxidant enzyme activities, and stomatal conductance under field conditions (Ibrahim 2025; Elkelish et al. 2026), and combining trehalose manipulation with salt-tolerant growth-promoting rhizobacteria aims to increase crop growth and yield (Kumawat et al. 2023). In this review, we lay the groundwork for future research to gain further insights into the possible molecular mechanisms behind trehalose and its intermediates functions in salinity tolerance, and to aid in their engineering for improving crop tolerance and yield in saline soils.

## Data Availability

No datasets were generated or analysed during the current study.
